# Acute Dietary Nitrate Supplementation and Exercise Performance in COPD: A Double-Blind, Placebo-Controlled, Randomised Controlled Pilot Study

**DOI:** 10.1371/journal.pone.0144504

**Published:** 2015-12-23

**Authors:** Katrina J. Curtis, Katie A. O’Brien, Rebecca J. Tanner, Juliet I. Polkey, Magdalena Minnion, Martin Feelisch, Michael I. Polkey, Lindsay M. Edwards, Nicholas S. Hopkinson

**Affiliations:** 1 NIHR Respiratory Biomedical Research Unit, Royal Brompton & Harefield NHS Trust and Imperial College, London, United Kingdom; 2 Centre of Human & Aerospace Physiological Sciences, King’s College London, London, United Kingdom; 3 Faculty of Medicine, Clinical and Experimental Sciences, University of Southampton, and Southampton NIHR Respiratory Biomedical Research Unit, Southampton General Hospital, Southampton, United Kingdom; Hunter College, UNITED STATES

## Abstract

**Background:**

Dietary nitrate supplementation can enhance exercise performance in healthy people, but it is not clear if it is beneficial in COPD. We investigated the hypotheses that acute nitrate dosing would improve exercise performance and reduce the oxygen cost of submaximal exercise in people with COPD.

**Methods:**

We performed a double-blind, placebo-controlled, cross-over single dose study. Subjects were randomised to consume either nitrate-rich beetroot juice (containing 12.9mmoles nitrate) or placebo (nitrate-depleted beetroot juice) 3 hours prior to endurance cycle ergometry, performed at 70% of maximal workload assessed by a prior incremental exercise test. After a minimum washout period of 7 days the protocol was repeated with the crossover beverage.

**Results:**

21 subjects successfully completed the study (age 68±7years; BMI 25.2±5.5kg/m^2^; FEV_1_ percentage predicted 50.1±21.6%; peak VO_2_ 18.0±5.9ml/min/kg). Resting diastolic blood pressure fell significantly with nitrate supplementation compared to placebo (-7±8mmHg nitrate vs. -1±8mmHg placebo; p = 0.008). Median endurance time did not differ significantly; nitrate 5.65 (3.90–10.40) minutes vs. placebo 6.40 (4.01–9.67) minutes (p = 0.50). However, isotime oxygen consumption (VO_2_) was lower following nitrate supplementation (16.6±6.0ml/min/kg nitrate vs. 17.2±6.0ml/min/kg placebo; p = 0.043), and consequently nitrate supplementation caused a significant lowering of the amplitude of the VO_2_-percentage isotime curve.

**Conclusions:**

Acute administration of oral nitrate did not enhance endurance exercise performance; however the observation that beetroot juice caused reduced oxygen consumption at isotime suggests that further investigation of this treatment approach is warranted, perhaps targeting a more hypoxic phenotype.

**Trial Registration:**

ISRCTN Registry ISRCTN66099139

## Introduction

Exercise limitation is a common symptom in patients with COPD. While the benefits of supplemental oxygen, at least in non-hypoxaemic subjects, on exercise performance remains uncertain[1], substantial skeletal muscle hypoxia is observed during exercise in both normal subjects and COPD patients whose oxygen saturation is maintained by the administration of supplemental oxygen[2]. Taken together with other data which suggest that anaerobic metabolism is frequent in patients with COPD[3, 4], a potential contributor to exercise limitation in COPD could be through the mechanism of tissue oxygen delivery, intramuscular oxygen transport or mitochondrial dysfunction. Thus approaches which reduce the oxygen cost of exercise might be of therapeutic benefit.

Both nutrient and oxygen delivery, as well as mitochondrial function (and thus oxygen consumption), are under regulatory control by the physiological signalling molecule nitric oxide (NO). NO is produced endogenously by the action of the NO synthase (NOS) family of enzymes via oxidation of the amino acid L-arginine. NO may also be produced by the reduction of exogenous dietary nitrate (NO_3_^-^), found at high levels in beetroot and green leafy vegetables[5], to nitrite (NO_2_^-^) and then further to NO in a NOS-independent manner[6].

Ingested nitrate is enterally absorbed into the blood, with approximately 25% of the circulating amount being taken up by the salivary glands (enterosalivary circulation). Nitrate is then excreted in saliva[7], and reduced from nitrate to nitrite via the nitrate reductase activity of oral commensal facultative anaerobic bacteria[8]. Salivary nitrite is subsequently swallowed and may enter the circulation directly as nitrite[8], or be further reduced to NO and absorbed following oxidation to nitrite or nitrate.

Various studies have reported benefits from dietary nitrate supplementation through the mechanism of a reduction in the oxygen cost of submaximal exercise in young, healthy individuals[9–13], and this has been associated with improved measures of exercise performance[9–12, 14]. The majority of studies have used beetroot juice as the nitrate source, the oxygen-sparing effect being dependent largely on nitrate itself since nitrate-depleted beetroot juice fails to elicit the same effect[11]. This effect has been shown to persist over at least 15 days of continual supplementation [12].

A beneficial effect of nitrate supplementation has also been demonstrated during exercise in hypoxic conditions[15], which is of interest for people with chronic respiratory disease. Two early studies suggested benefit in patients with COPD [16, 17], although both studies were limited by the placebo preparation selected, and other groups have failed to reproduce these findings using a robust placebo[18, 19]. However, in the two latter studies the dose of nitrate administered immediately prior to testing was below that which has been shown in health to induce improvement in exercise performance[6], and in one case testing was performed only one hour post dosing[19] when nitrite levels may not yet have peaked. Thus these findings need further investigation with a higher dosing regimen given at an appropriate time interval prior to exercise testing, particularly since the studies failed to show any effect on blood pressure[18, 19]. The aim of this study was to investigate the effects of a higher dosing regimen of nitrate, in the form of beetroot juice, on exercise performance in COPD with concomitant measurements of pulmonary oxygen uptake and with comparison to a robust placebo preparation.

## Materials and Methods

### Patient selection

All participants provided written informed consent prior to enrolment in the study, which was approved by the Bromley Research Ethics Committee (reference 13/LO/0372) and conducted in line with the principles of the Declaration of Helsinki. COPD patients of GOLD stage II-IV[20] were considered for inclusion and recruited through outpatient clinics at the Royal Brompton Hospital and through public outreach events including World COPD Day. Recruitment started on 24^th^ June 2013 and was completed on 28^th^ April 2014, with follow-up completed on 3^rd^ June 2014. This study was registered prospectively on a publicly accessible database (www.iscrn.com/ISRCTN66099139). The authors confirm that all ongoing and related trials for this intervention are registered.

Exclusion criteria for the study included patients taking nitrate-based medications or phosphodiesterase V inhibitors, those with other medical conditions likely to benefit from nitrate supplementation (including ischaemic heart disease and peripheral vascular disease), and patients on long-term oxygen therapy or antibiotic therapy (whether on an acute or prophylactic basis). Those patients considered clinically unstable (within one month of pulmonary exacerbation) or suffering from hypotension or significant renal impairment (eGFR<50ml/min/1.73m^2^) were also excluded from the study.

### Study design and randomisation

The study was a double-blind, cross-over, placebo-controlled trial. Allocation of the order of beverages was performed using a computer-generated randomisation in blocks of 4, produced by an independent statistician with consecutive numbers linked to preparations of placebo or active treatment. The researcher responsible for enrolment and outcome measurements remained separate from the randomisation process and remained blinded throughout the study and during data analysis. The principal investigator who was not involved in any patient visits held the randomisation list and indicated the patient assignment order.

We hypothesised that nitrate supplementation would increase time to exhaustion during symptom-limited cycle ergometry at 70% peak workload as assessed previously using an incremental, maximal, symptom-limited cardiopulmonary exercise test. Secondary outcome measures included plasma nitrate and nitrite levels, blood pressure response, area under the VO_2_ curve to isotime, and plasma metabolomics.

### Intervention

140ml of BEET-IT Sport Stamina Shot (James White Drinks, Ipswich, UK) containing 0.8g nitrate and 140ml of a matched placebo of beetroot juice specifically depleted of nitrate were used as the active and placebo preparations, respectively. The placebo preparation was identical in appearance and taste[11], and also produced beeturia. The dose chosen was intended to provide a bolus of nitrate (12.9mmoles) exceeding that necessary to reduce oxygen consumption during submaximal exercise in young healthy subjects[6], and was selected as a convenient dosing method (2x 70ml bottles) which was readily acceptable and easily ingested.

Subjects were requested to avoid foods naturally high in nitrate in the 48hour period prior to the dosing visit and were asked to match food and caffeine consumption on the two days of testing. Subjects were additionally asked to avoid use of mouthwash and chewing gum in the 48hour period prior to testing as this has previously been shown to reduce oral bacterial nitrate reductase activity[21]. Subjects were also advised to avoid heavy exercise in the 24 hours prior to each dosing visit.

### Study conduct

All data were collected at the NIHR Respiratory Biomedical Research Unit at the Royal Brompton and Harefield NHS Foundation Trust. Following successful screening the subjects attended for measurement of baseline characteristics during which several parameters were recorded including vital signs, anthropometric and bioimpedance measurements, quality of life assessment, full pulmonary function testing, quadriceps maximal volitional contraction, physical activity monitoring, and an incremental maximal symptom-limited cycle ergometry test was performed.

On the dosing visits the subjects attended on the day of testing in the morning; after a 10 minute rest period blood pressure measurements and baseline plasma samples were obtained, after which the patients were dosed. 3 hours later both blood pressure measures and blood sampling was repeated prior to endurance cycle ergometry at a constant work rate (70% peak workload achieved on prior maximal incremental exercise testing). The cycle test was conducted at the same time on each occasion. Further blood draws were taken at peak exercise. After a minimum wash-out period of 7 days the protocol was repeated with the crossover beverage.

### Blood pressure, anthropometrics and fat fee mass measurements

Blood pressure was measured using an automated blood pressure monitor (Omron M6, Omron Healthcare Europe, Hoofddorp, Netherlands). Measurements were performed in the seated position with the subject’s arm supported. A mean of three measurements was recorded. Height (cm) without shoes was measured using a wall mounted measure, and weight (kg) measured using standardised scales.

Fat free mass (FFM) was determined using bioelectrical impedance analysis by measuring the electrical resistance between the wrist and ankle using a Bodystat 4000 device (Bodystat, Isle of Man, UK). Subjects lay supine for 10 minutes prior to measurement. Electrodes were placed on the dominant hand and foot, at 2cm proximal to the carpometacarpal joint and the ulnar level of the wrist on the dorsal aspect of the hand, and at 2cm proximal to the carpometatarsal joint and level of the malleoli of the ankle respectively. The value obtained is dependent on body water content and based on this FFM can be calculated using a disease specific regression equation[22]. Fat Free Mass Index (FFMI) was calculated by dividing FFM by height in metres squared (kg/m^2^). Individuals with a FFMI below 16 kg/m^2^ in males and 15 kg/m^2^ in females were considered to have evidence of FFM depletion[22].

### Physical activity measurement

Patients were asked to wear a physical activity monitor (SenseWear Pro Armband, Bodymedia, Pittsburg, USA) continually for a week that incorporates a biaxial accelerometer and energy expenditure measurements[23]. The armband was placed over the body of the triceps muscle of the right arm and the subject asked to wear it continuously except when bathing. Data was analysed over a period of 5 days including 2 weekend days.

### Quadriceps strength

Maximum isometric quadriceps force (QMVC) was measured using the technique of Edwards et al.[24]. Prior to each measurement calibration was performed using a standardised weight. Subjects were seated with the leg flexed at an angle of 90° relative to the edge of the couch. The right leg was tested unless it was not feasible to do so. An inextensible strap was placed around the subject’s ankle and connected to a strain gauge. The maximum force generated and sustained for at least 1 second during at least five contractions with vigorous encouragement was measured (recorded in kg). A minimum of 30 seconds was allowed between each effort. Output from the strain gauge was processed as a digital output by a Powerlab recording unit and analysed using LabChart software (AD Instruments, Oxfordshire, UK) sampling at 10kHz. The percentage predicted QMVC was determined according to regression equations based on age, gender and FFM[24, 25].

### Pulmonary function testing

Measurements were made in the lung function department of the Royal Brompton Hospital according to international guidelines and with rigorous quality assurance in place with a Jaeger master lab system (CompactLab system, Jaeger, Wurzburg, Germany). Spirometry, gas transfer and plethysmographic lung volumes were also measured in accordance with European Respiratory Society (ERS)/ American Thoracic Society (ATS) guidelines[26–28]. Standardised lung function testing reference equations were based on the European Coal and Steel Community (ECSC) reference values[29]. Capillary blood gas samples were taken at rest.

### Health-related quality of life measures

Subjects completed the St George Respiratory Questionnaire for COPD patients (SGRQ-C) and the COPD Assessment Test (CAT). Each is validated in assessing health-related quality of life in COPD [30, 31].

### Cycle ergometry testing

A symptom limited incremental exercise test was performed at baseline on a bicycle ergometer (Ergoselect 100, Ergoline, Bitz, Germany) with metabolic measurements collected using a mouthpiece (Masterscreen CPX metabolic cart, CareFusion, Basingstoke, UK) and analysed using JLAB software (JLAB Lab Manager version 5.32, Jaeger, CareFusion, Basingstoke, UK). Following a 2 minute rest period and 2 minute free cycling workload increased by 5 Watts every 30 seconds with subjects being asked to maintain a speed of 60–70 revolutions per minute. Measurements were taken of peak workload, VO_2_, VCO_2_, minute ventilation, respiratory rate and tidal volume. Peak workload was defined as the greatest workload that the subject was able to maintain for a 30 second period.

To evaluate the effects of the intervention on cardiorespiratory fitness and skeletal muscle function endurance cycle ergometry testing was performed 3 hours following dosing at 70% peak workload achieved on incremental cycle ergometry. The endurance time was calculated as the time from the beginning of loaded cycling to the point of test cessation due to symptom limitation. Breath-by-breath data was obtained and rolling 8-breath averages were used in the isotime analysis (with isotime being defined as the duration of the shorter of the two exercise tests).

### Plasma nitrate/nitrite analysis

All plasma samples (baseline, 3 hours post dosing and peak exercise) were obtained by venesection in lithium heparin tubes and immediately spun for 10 minutes at 520g at 4°C. The resulting supernatant was pipetted into 2ml polypropylene cryotubes, taking care to avoid any contamination from the buffy coat containing the white cells/ platelets atop the erythrocyte pellet, and immediately frozen at -80°C.

Nitrate and nitrite plasma levels were assessed using a post-column diazo coupling reaction (Griess reaction) in combination with high-performance liquid chromatography (HPLC) (Eicom NOx analyser, ENO-20, San Diego, USA)[32]. Nitrate and nitrite were first separated on the analytical column. Nitrite then reacts with the Griess reagent generating a red diazo compound, the absorbance of which was measured at 540nm using a spectrophotometric detector. Nitrate passing through the on-line reduction column was reduced to nitrite before undergoing the same diazo coupling reaction. The peak areas of absorption of solutions of know standard concentrations were then compared to those produced by the test samples to provide measures of plasma nitrate and nitrite.

### Plasma nuclear magnetic resonance spectroscopy analysis

Plasma samples were defrosted at room temperature and centrifuged at 16,000g for 10 minutes. An aliquot of 300μl was then mixed with 300μl of NMR buffer (double distilled water containing 8% deuterium oxide (D_2_O) for the magnetic lock). The resulting mixture was transferred to 5mm NMR tubes within a 96-tube rack. ^1^H-NMR spectra of plasma samples were obtained using a Bruker Avance III 700 MHz spectrometer (Bruker Biospin, Karlsruhe, Germany) at the operating ^1^H frequency 700.2 MHz with a temperature of 298K. A Carr-Purcell-Meiboom-Gill (CPMG) pulse sequence (Bruker experiment: cpmgpr1d) was applied to better visualise low molecular weight metabolites. A spin relaxation delay of 76ms was used and a total of 32 scans were acquired.

Spectra were processed and analysed as described previously[33]. An exponential window function was applied with a line broadening of 0.3 Hz prior to Fourier transformation. All chemical shifts were manually referenced to formate (δ = 8.44 ppm).

### Data analysis and statistics

We studied a convenience sample of 25, offering a good chance of identifying a clinically meaningful effect. Data are presented as mean ± standard deviation and were analysed using GraphPad Prism version 5.0 for Windows (GraphPad Software, San Diego, California, USA). Repeated measures analysis of variance (ANOVA) was utilised to compare plasma nitrate metabolites between treatment conditions and at each specific time-point. To compare oxygen consumption between study conditions, individual cardiopulmonary exercise test data periods were expressed as percentiles of isotime (the duration of the shorter of the two tests) and the individual responses grouped to allow analysis of VO_2_ against percentage of isotime (plotted at the midpoint of each 10^th^ percentile of isotime). The area under this curve was assessed for each subject and the two conditions, then compared using a paired t-test. Resting and isotime measurements between treatment conditions were compared using paired t-tests, or the appropriate non-parametric test for data that was not normally distributed. A p-value <0.05 was considered to be statistically significant.

## Results

### Subjects

**Fig 1:**
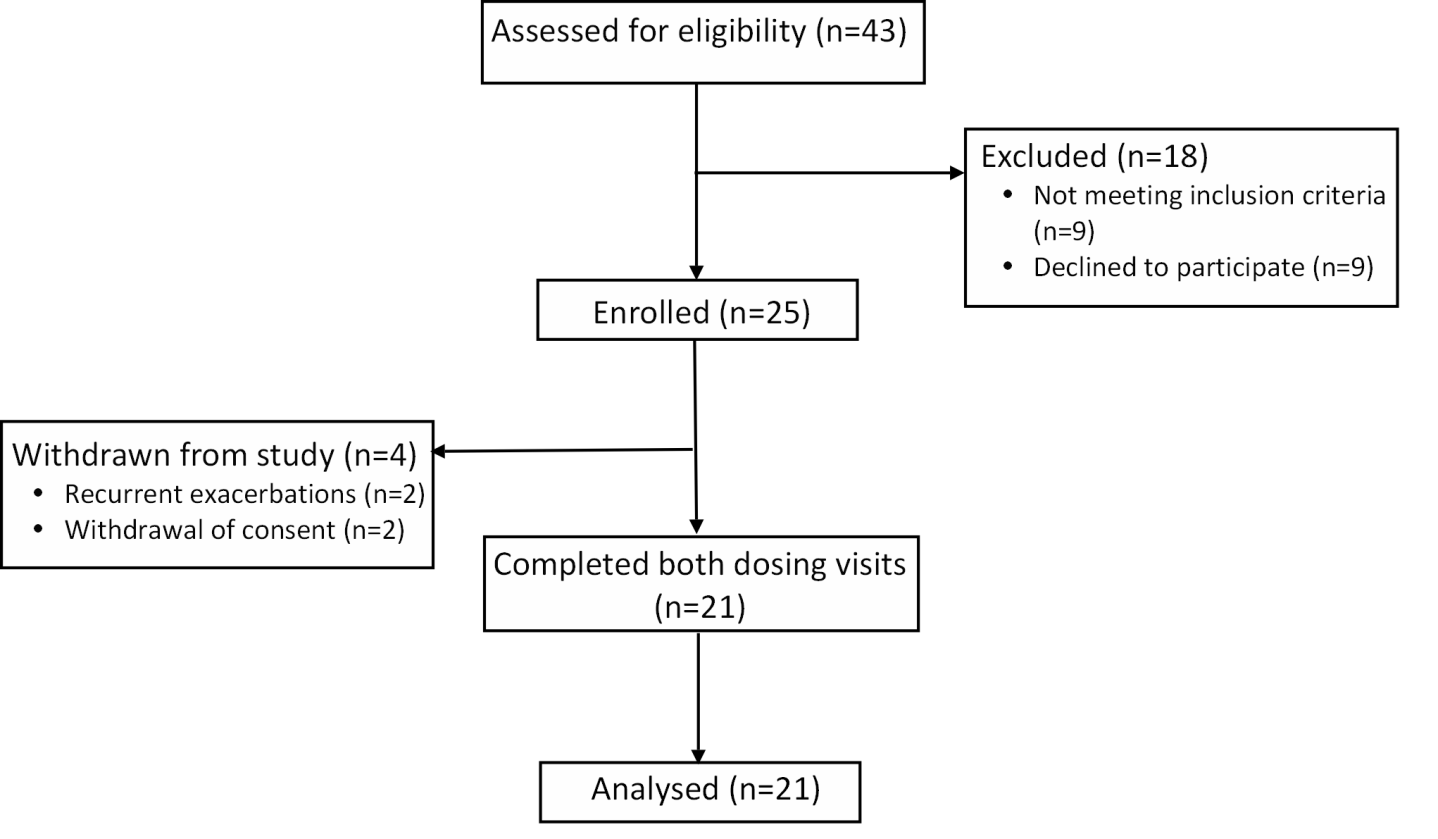
CONSORT recruitment diagram for enrolment and study completion.

25 patients were enrolled into the study, of whom 21 completed the full study protocol (Fig 1). Recruitment started in June 2013 and the trial completed in June 2014 when the 25 enrolled patients had either completed or been withdrawn. The baseline characteristics of the participants are presented in Table 1. Patients were 100% compliant with the dosing protocol with no adverse effects noted except beeturia.

**Table 1:** Demographic and baseline clinical characteristics of the subjects (n = 21).

Measurement	Mean (SD)
Sex (♂:♀)	16:5
Age (years)	68 (7)
Systolic BP (mm Hg)	137 (19)
Diastolic BP (mm Hg)	79 (7)
Smoking PYH	40 (29)
MRC dyspnoea score	2 (1)
CAT score	14 (7)
SGRQ-C total	35.37 (13.00)
BMI (kg/m^2^)	25.2 (5.5)
FFM (kg)	48.9 (8.4)
FFMI (kg/m^2^)	16.9 (2.2)
QMVC (kg)	37.8 (10.7)
QMVC % predicted	87.8 (19.3)
Average daily step count	6669 (4436)
Average PAL	1.50 (0.20)
FEV_1_ (L)	1.33 (0.58)
FEV_1_% predicted	50.1 (21.6)
TLCO_c_ % predicted	54.1 (19.3)
RV % predicted	167 (53)
TLC % predicted	119 (14)
RV/TLC ratio (%)	52.3 (8.7)
PaO_2_ (kPa)	10.7 (1.2)
Peak power on cycle (watts)	71.9 (30.8)
Peak VO_2_ (ml/min/kg)	18.0 (5.9)

Data shown are mean (SD). Abbreviations: BP–blood pressure; PYH–pack year history; MRC–Medical Research Council; CAT–COPD assessment test; SQRC-C–St. George’s respiratory questionnaire for COPD; BMI–body mass index; FFMI–fat free mass index; QMVC–quadriceps maximal volitional contraction; PAL- physical activity level; FEV_1_ –forced expiratory volume in 1 second; TLCO_c_−carbon monoxide diffusing capacity; RV–residual volume; TLC–total lung capacity; VO_2_ –pulmonary oxygen uptake.

### Venous nitrate and nitrite measures

**Fig 2:**
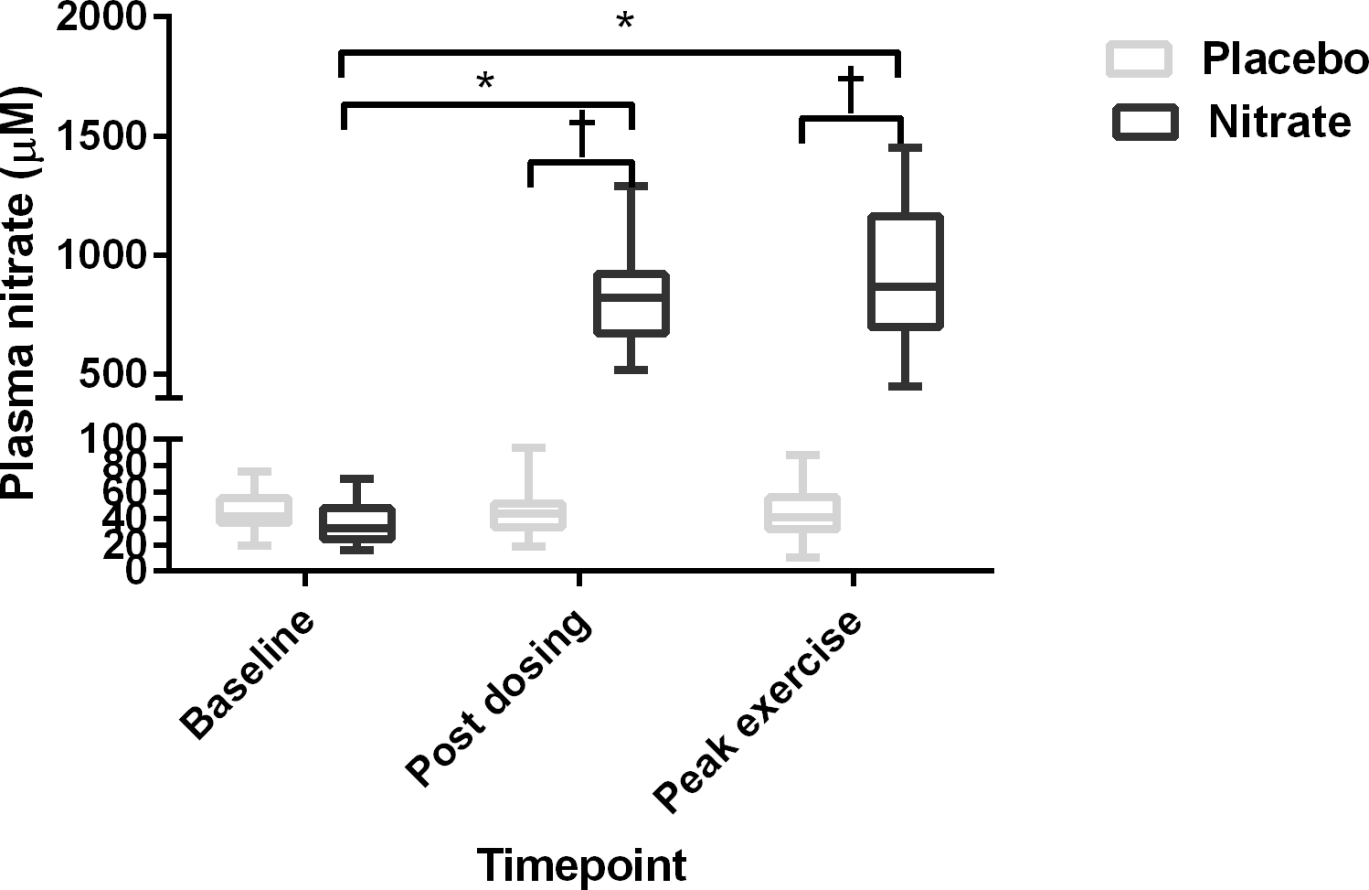
Alterations in plasma nitrate following dosing. Plasma nitrate concentrations prior to (‘baseline’) and following consumption of a nitrate-rich or placebo beverage. ‘Post dosing’ indicates a time point 3 hours following consumption which was immediately prior to cardiopulmonary exercise testing. ‘Peak’ was a time point at the point of exhaustion during endurance cycle ergometry testing. Repeated measures analysis of variance (ANOVA) was used to compare plasma nitrate metabolites between treatment conditions and at each specific time-point; *significantly different from baseline, p<0.0001; †significantly different from placebo group, p<0.0001.

Venous plasma nitrate measurements at all timepoints are shown in Fig 2. Following dosing with nitrate-rich beetroot juice plasma nitrate levels increased substantially (37.0 ± 16.4μM baseline vs. 820.2 ± 187.7μM post dosing; p<0.0001) and remained significantly elevated at peak exercise (917.1 ± 291.6μM; p<0.0001). There were no significant changes in plasma nitrate concentrations following dosing with the placebo preparation (45.7 ± 15.8μM baseline vs. 45.3 ± 16.5μM post dosing; p>0.99). Plasma nitrite levels were below the quantifiable limit (0.2μM) in all but three patients at baseline and remained so in the placebo condition but increased following nitrate supplementation to 1.57 ± 0.98μM and remained elevated at peak exercise (1.37 ± 0.65μM).

### Endurance exercise time

**Fig 3:**
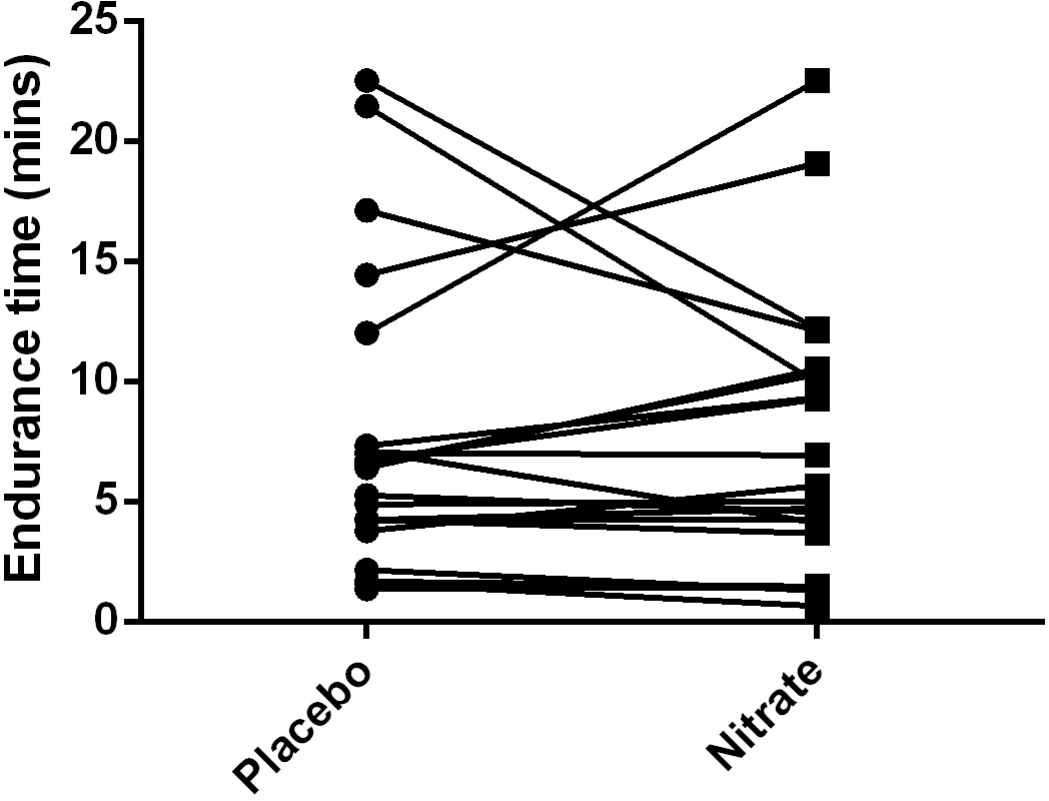
Endurance time during cycle ergometry at 70% maximal workload measured in the placebo and nitrate-rich beetroot juice dosing conditions. A Wilcoxon test was used to compare median values in the two treatment groups; no significant difference was found.

The median endurance time during cycle ergometry performed at 70% maximum workload was not significantly different between the treatment groups (5.65 (3.90–10.40) nitrate vs. 6.40 (4.01–9.67) minutes placebo; p = 0.50). The individual responses are depicted in Fig 3. We also tested for an order effect, but none was present (the change in endurance time if nitrate supplementation was applied first was -2.03±4.64 minutes, compared to 1.92±4.16 minutes if placebo was administered first; p = 0.13).

### Blood pressure responses

**Fig 4:**
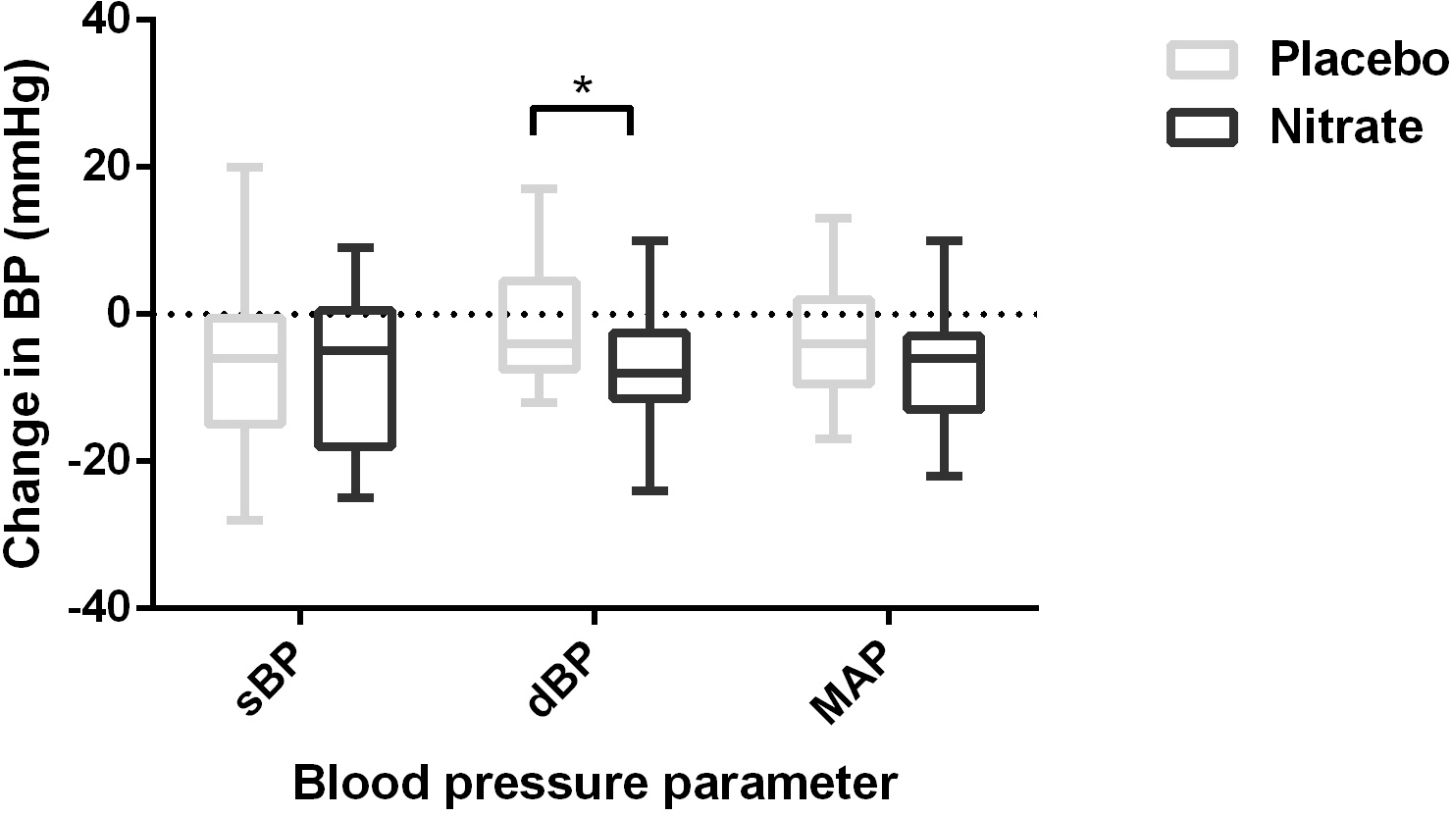
Blood pressure parameters following dosing. Alterations in blood pressure parameters (systolic blood pressure sBP, diastolic blood pressure dBP and mean arterial pressure MAP) relative to presupplemented baseline 3 hours following dosing with nitrate-rich beetroot juice or placebo preparation. Paired t-tests were used to compare blood pressure parameters between treatment groups; *significantly different from placebo, p<0.01.

The changes in resting blood pressure parameters following ingestion of nitrate-rich beetroot juice and placebo are shown in Fig 4. The reduction in systolic blood pressure after dosing between treatment conditions was not significantly different, however, the reduction in diastolic blood pressure was greater in the nitrate supplemented group (7±8 nitrate vs. 1±8mmHg placebo; p = 0.008). There was a trend towards greater lowering of the mean arterial pressure following nitrate supplementation, although this was not statistically significant (7±8 nitrate vs. 3±8mmHg placebo; p = 0.07).

### VO_2_ curves to isotime

At rest there was no significant difference in minute ventilation or VO_2_ following dosing with either nitrate-rich beetroot juice or placebo (Table 2). At isotime (i.e. the longest time of equivalent exercise completed under either study condition) both pulmonary VO_2_ and minute ventilation were lower in the nitrate supplemented condition than placebo (Table 2). There was a numerical improvement in the power/VO_2_ relationship (P/O ratio) in the nitrate supplemented condition, although this was not statistically significant.

**Table 2:** Rest and isotime analysis of the cardiopulmonary exercise test parameters.

Parameter	Timepoint	Placebo	Nitrate-rich beetroot juice	Difference	p value
HR (bpm)	Rest	85 (11)	87 (11)	2 (7)	0.063
HR (bpm)	Isotime	122 (17)	121 (20)	-1 (10)	0.30
BF (breaths/min)	Rest	16 (4)	17 (4)	1 (3)	0.095
BF (breaths/min)	Isotime	32 (8)	32 (5)	0 (7)	0.48
VT (L)	Rest	0.93 (0.43)	0.87 (0.25)	-0.06 (0.26)	0.33
VT (L)	Isotime	1.44 (0.39)	1.40 (0.47)	-0.04 (0.18)	0.12
VE (L/min)	Rest	13.27 (3.90)	13.37 (3.95)	0.10 (2.29)	0.34
VE (L/min)	Isotime	43.85 (13.94)	41.88 (14.85)	-1.97 (4.52)	0.029*
VO_2_ (ml/min/kg)	Rest	4.5 (1.2)	4.4 (1.3)	-0.1 (0.9)	0.32
VO2 (ml/min/kg)	Isotime	17.2 (6.0)	16.6 (6.0)	-0.6 (1.48)	0.043*
VCO_2_ (ml/min/kg)	Rest	4.2 (1.2)	4.2 (1.3)	0.0 (0.82)	0.49
VCO2 (ml/min/kg)	Isotime	17.1 (6.0)	16.7 (6.3)	-0.4 (1.74)	0.14
Power/VO_2_ ratio (W/ml/min/kg)	Isotime	2.92 (0.73)	3.03 (0.73)	0.11 (0.29)	0.053
SpO_2_ (%)	Rest	95 (2)	96 (2)	0 (2)	0.26
SpO_2_ (%)	Isotime	92 (4)	93 (4)	1 (2)	0.15
Oxygen pulse (ml/beat)	Rest	3.8 (1.0)	3.6 (0.9)	-0.2 (0.69)	0.12
Oxygen pulse (ml/beat)	Isotime	10.0 (2.6)	9.7 (2.3)	-0.3 (1.2)	0.09
Relative oxygen pulse (ml/beat/kg)	Rest	0.053 (0.0138)	0.051 (0.014)	-0.002 (0.010)	0.15
Relative oxygen pulse (ml/beat/kg)	Isotime	0.143 (0.039)	0.138 (0.036)	-0.005 (0.014)	0.043*

Data are shown as mean (SD)

*p<0.05.

Abbreviations: HR—heart rate; BF–breathing frequency; VT–tidal volume; VE–minute ventilation; VO_2_ –pulmonary oxygen uptake; VCO_2_—exhaled carbon dioxide production; SpO_2_ –peripheral arterial oxygen saturation.

**Fig 5:**
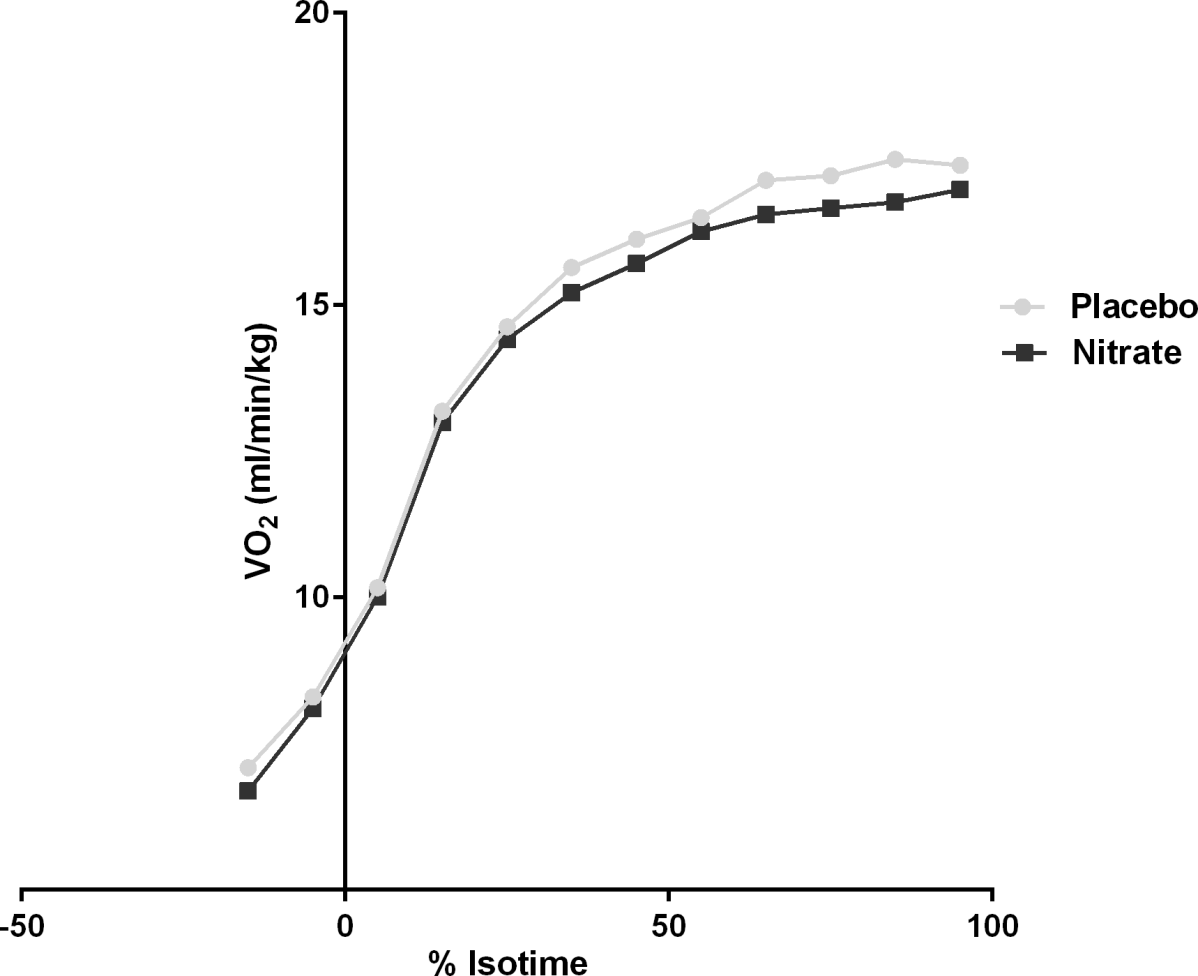
Isotime VO_2_ analysis. The graph represents the VO_2_ at each 10^th^ percentile of isotime with 0% representing the initiation of loaded cycling at 70% maximal workload achieved on incremental cycle ergometry. The area under the curves to isotime was compared using a paired t-test showing significant separation of the curves representing the two treatment conditions (p = 0.027).

**Fig 6:**
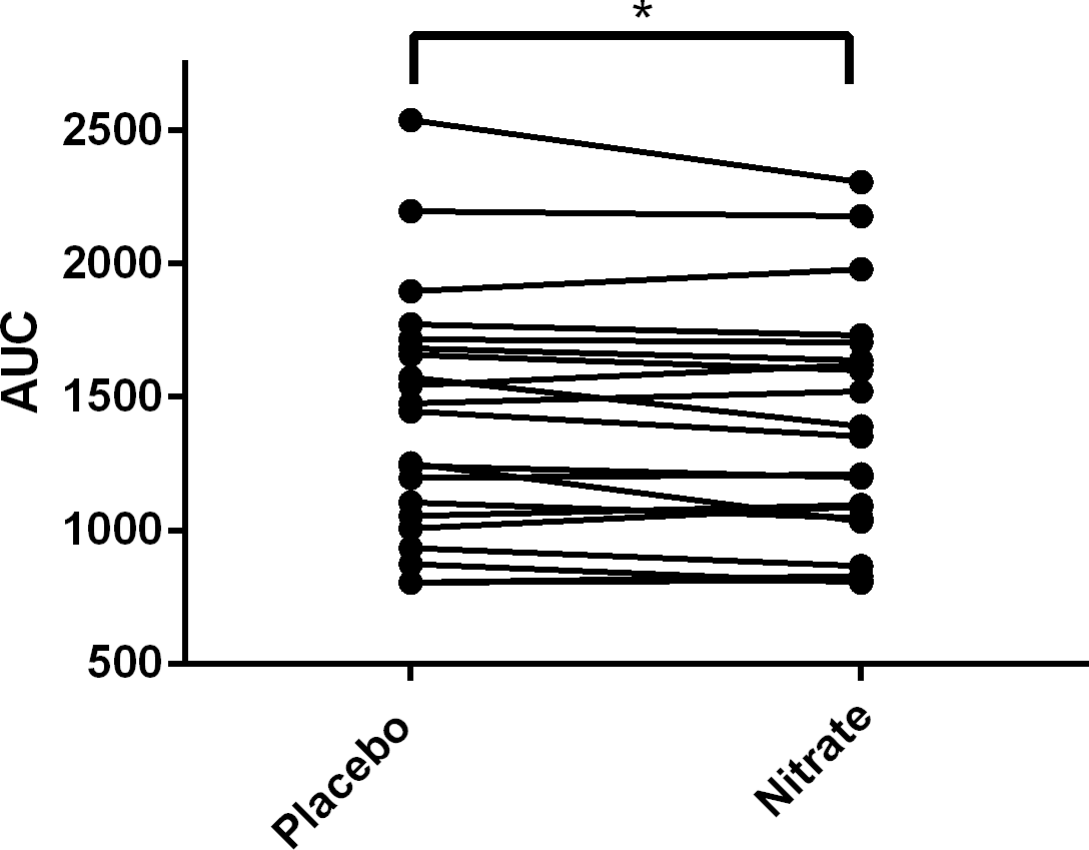
Individual responses for area under the curve (AUC) to VO_2_ isotime. There was a significant difference between the two treatment conditions with a reduction in the area of the curve to VO_2_ isotime in the nitrate supplemented condition.

VO_2_ curves to isotime revealed a significant separation of the curves between the two treatment conditions (Fig 5). The area under the curve to VO_2_ isotime was also calculated for each subject to enable a comparison of the two treatment conditions (Fig 6).

### Plasma NMR spectroscopy analysis

**Fig 7:**
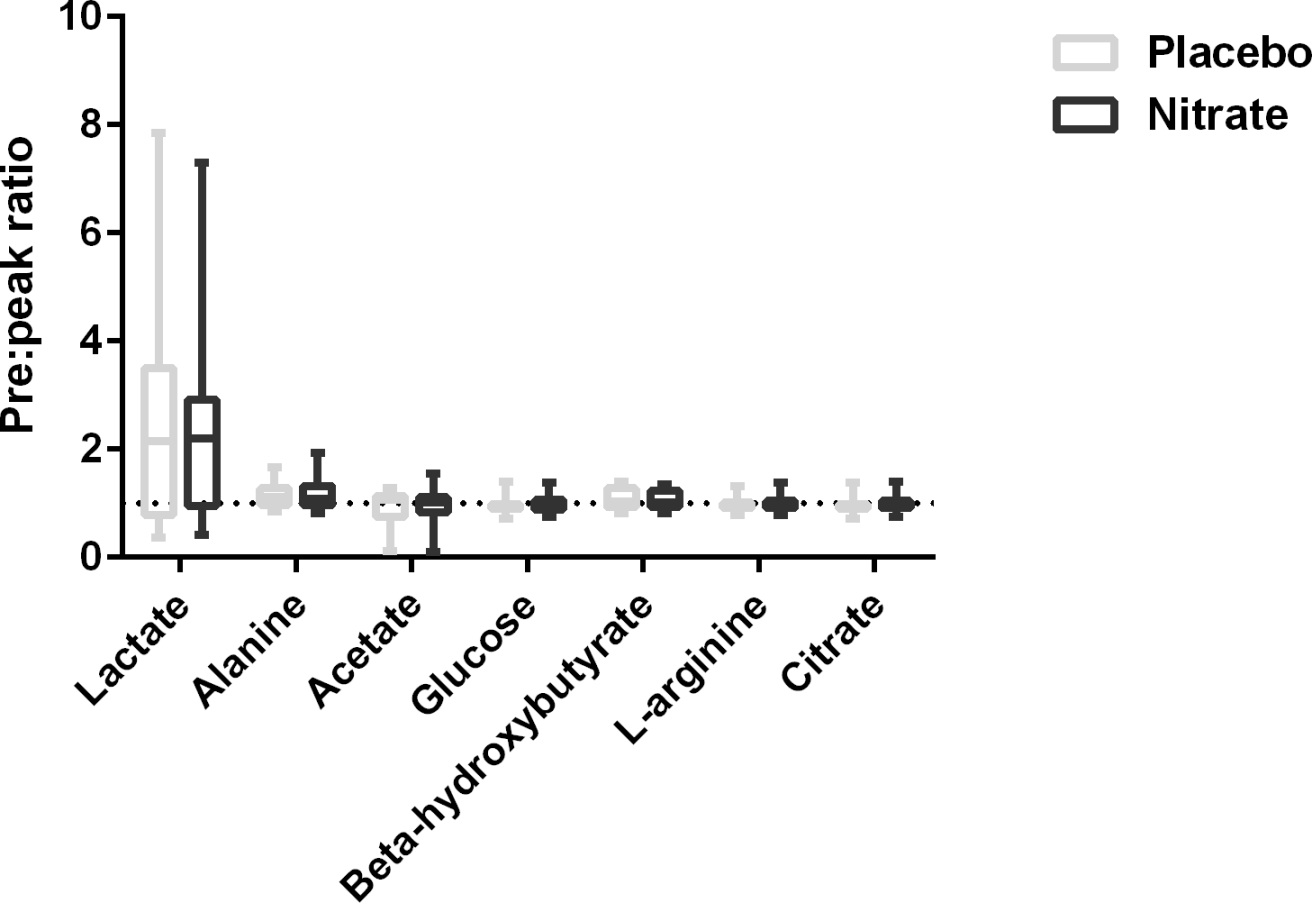
Alteration in plasma metabolomics during endurance cycle ergometry. Values are expressed as a ratio of peak exercise:pre exercise, with a value of 1 indicative of no change. Paired t-tests were utilised to compare metabolite levels between treatment conditions, with no significant difference seen for any metabolite analysed.

Exercise related plasma metabolites showed no difference when comparing pre and peak exercise values under the two treatment conditions (Fig 7).

## Discussion

Our trial, in patients with COPD, failed to demonstrate an improvement in endurance exercise performance following acute nitrate dosing in the form of beetroot juice. However, nitrate supplementation was biologically active, causing reductions in resting diastolic blood pressure and oxygen consumption at isotime during exercise, which may warrant further investigation.

### Critique of the method

Adequate nitrate supplementation was confirmed by the observation of a 22-fold rise in plasma nitrate within 3 hours. Baseline plasma nitrate levels were similar to those of young recreationally active individuals[6, 34] and, whilst we were concerned that possible age related changes in digestion, enterosalivary circulation and oral commensal bacteria might impair nitrate delivery, the plasma nitrate levels achieved exceeded even those achieved by administration of a higher dose of nitrate in healthy young individuals[6]. Secondly, we observed a statistically significant reduction in diastolic blood pressure as previously noted in healthy individuals[35, 36], seniors with peripheral vascular disease[37], and COPD patients[16, 17]. One previous study has failed to show a reduction in blood pressure parameters following acute nitrate dosing in COPD[18]. However that study, which also failed to show an effect on exercise performance, used a lower treatment dose of nitrate and achieved plasma nitrate levels that were 4-fold lower than our dosing regimen (215±84μM vs. 820±288μM). Previous work has established that both reduction in blood pressure and post-dose plasma nitrate levels are dose-dependent[6]. Thus an important conclusion of the current study is that a single higher dose of nitrate is both safe and biologically active in COPD.

The plasma nitrite levels fell below the limit of quantifiability in the majority of baseline samples. Part of the explanation for this, besides a small interfering peak apparently originating from the beetroot juice, is that samples were not pre-treated with a thiol-blocking agent prior to centrifugation; such treatment ensures there is no flux of nitrite across blood cell membranes, which may lower plasma concentrations. However, high levels of plasma nitrite were readily measurable following nitrate dosing.

We failed to observe an improvement in exercise performance following nitrate dosing, despite a reduction in the oxygen cost of exercise. There are several possible explanations for this. Firstly, this could be due to statistical power and variation in endurance time. The variability in endurance time at 70% maximal workload on endurance cycle ergometry was beyond what we have observed in previous studies in a similar population[38]. In part this may be explained by day-to-day symptom variability in COPD[39], and might thus be an expected feature of studies in this condition using this outcome measure as has been reported by other groups studying nitrate supplementation in COPD[16, 17, 19]. A trial run might have reduced this variability, although it is important to note no order effect was identified. Studies using cycle ergometry methodology and agents of proven efficacy (for example bronchodilators) have tended to have larger sample sizes than ours[38, 40, 41]. Certainly, significant caution is needed when interpreting the results of non-blinded studies in this field, and emphasises the importance of using nitrate-depleted beetroot juice (which also causes beeturia) as a placebo[16, 17].

Secondly, it may simply be the case that this small improvement in the oxygen cost of exercise does not impact on the rate limiting parameters in exercise in ventilatory limited patients. Thirdly, we may have studied the wrong patient group. By design we did not include patients on oxygen supplementation but it may be the case that it is this specific patient phenotype where a reduction in the oxygen cost of exercise produces the greatest benefit.

### Significance of the findings

While it is possible that a larger sample size might have given a positive result this seems relatively unlikely given exercise time was also numerically shorter in the treatment group. However, the observation (confirmed by the fall in blood pressure) that nitrate supplementation is biologically active and associated with a measurable reduction in oxygen consumption is of interest. Cardiovascular comorbidities are common in patients with COPD, so an intervention that lowers blood pressure may produce clinical benefit outside of a direct effect on exercise performance.

Some prior data are available regarding nitrate supplementation with beetroot juice in COPD. An improvement in endurance cycle ergometry exercise time has been previously noted in a randomised cross-over study[16]. However, we believe that caution is needed in interpreting this result, partly because a matching placebo beverage (i.e. one which caused beeturia) was not used. This study as noted by the authors, also noted inter-individual variability in endurance time despite the use of a familiarisation constant work rate exercise test, and appropriate adjustment if the endurance time was outside of the 4–10 minute range. In fact, some individual responses were of a greater magnitude than could be explained by a plausible biological mechanism, thus skewing the overall data for endurance time. Despite this, in line with our own study, a similar reduction in pulmonary oxygen uptake at isotime was noted (of the order of 0.7ml/min/kg), although this failed to reach statistical significance (p = 0.099) most likely due to under-powering[16]. This lends support to our own findings regarding the influence of nitrate supplementation on the oxygen cost of exercise.

Two further studies in COPD (both reported after we started our study) used a robust placebo but either failed to show a positive effect on oxygen consumption[18] or did not measure this variable[19], and both showed no improvement in exercise performance[18, 19]. Effects on oxygen consumption have previously been shown to be dose-dependent[6], and thus these negative findings may be due to the use of an insufficient dose of nitrate. Consideration should also be given to the fact that there are recognised ‘responders’ and ‘non-responders’ to nitrate supplementation when studying athletic populations[42, 43]. Whilst the lack of response in elite athletes may in part be explained by intrinsic high levels of nitrate metabolites through both exercise induced upregulation of NOS activity[44] and the selection of a diet rich in nitrate, the fact that within that group there is variability in response indicates that other unrecognised factors are likely at play[42, 43]. Our own study is too small to enable effective subanalysis but it should remain a focus of future studies to consider factors that may affect responsivity to nitrate loading, allowing those subjects most likely to benefit from nitrate supplementation to be both recognised and targeted.

The reduction of VO_2_ at absolute isotime in our study is of a similar magnitude (3–4%) to that recorded in healthy individuals[6]. The cause of this observation is unclear, but of interest. Several potential modes of action of nitrate have been proposed including alterations to skeletal muscle blood flow[45], improved efficiency of the mitochondrial oxidative phosphorylation[46], effects on energy requiring processes in skeletal muscle[47, 48], alterations to mitochondrial biogenesis[49], blood viscosity[50] and NOS substrate availability[51]. No significant changes were noted in any of the metabolites identified in the NMR spectroscopy array, and although lactate rose appropriately following the exercise challenge this was of equal magnitude in both conditions, suggesting, as in health, that nitrate does not promote anaerobic metabolism[9].

Use of phosphorus-31 NMR spectroscopy have suggested that nitrate supplementation acts, at least in part, via reduced ATP cost of muscle force production[9]. Separately we have recently demonstrated a trend towards longer ADP recovery times in COPD patients preselected for quadriceps dysfunction[3]. The generation of ATP is dependent on the generation and maintenance of the electrochemical proton gradient. However, not all of this membrane potential is eventually coupled to ATP production due to proton leakage across the inner mitochondrial membrane, through uncoupling proteins and the adenine nucleotide translocase. Larsen *et al*.[46] demonstrated ex vivo that following 3 days of nitrate supplementation harvested human skeletal muscle mitochondria increased oxidative phosphorylation efficiency, correlating with a 3% reduction in whole-body oxygen consumption during submaximal workload exercise[46]. Future studies are clearly required to inform the mechanistic action of nitrate to allow the exploration of pharmacological therapies that may exploit the relevant pathways.

Although our data do not support a role for general use of nitrate supplementation using beetroot juice in this setting we believe, because of the observation of reduced oxygen consumption, that targeting specific phenotypes in COPD may lead to the identification of therapeutic benefits. Thus, for example, we are now conducting a trial to investigate nitrate supplementation as an adjunct to pulmonary rehabilitation (www.controlled-trials.com/ISRCTN27860457), powered to detect an improvement in incremental shuttle walking distance. Other possible feasibility studies could be those in patients stratified for lactataemia (indicating anaerobic metabolism) during exercise, or those with either systemic or marked local hypoxia (for example detected using near-infrared spectroscopy[2]).

In conclusion, acute administration of 0.8g nitrate in the form of beetroot juice failed to improve endurance exercise cycle capacity compared with placebo, but the reduction in oxygen consumption during exercise in COPD subjects and the reduction observed in resting diastolic blood pressure confirm a biological action at this dose. Several studies have now confirmed the blood-pressuring lowering properties of nitrate administered as beetroot juice in COPD, and as comorbidities are common in this population this may be an important effect. Since the intervention is safe and inexpensive further studies are warranted to establish whether it has a therapeutic role in specific COPD phenotypes.

## Supporting Information

S1 CONSORT Checklist (DOC)

S1 Trial Dataset (XLS)

S1 Trial Protocol (DOCX)

## Abbreviations

COPD: chronic obstructive pulmonary disease
FFM: fat free mass
NMR: nuclear magnetic resonance
NO: nitric oxide
NOS: nitric oxide synthase
