# Supplementary material for: Acute Dietary Nitrate Supplementation and Exercise Performance in COPD: A Double-Blind, Placebo-Controlled, Randomised Controlled Pilot Study
Source: PLoS One. 2015 Dec 23;10(12):e0144504. doi: 10.1371/journal.pone.0144504 (PMC4689520; doi:10.1371/journal.pone.0144504)
Supplement: S1 Trial Protocol — (DOCX) [file pone.0144504.s003.docx]

#
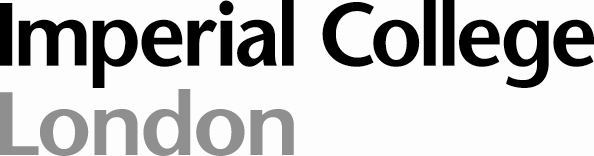

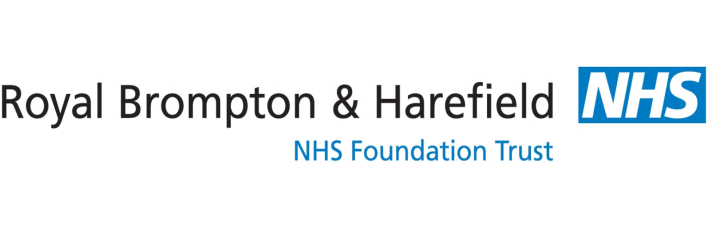


EDEN-EPIC

Effect of dietary nitrate supplementation on exercise performance in COPD

V1 13/02/2013

MAIN SPONSOR or co-sponsors: Imperial College London

FUNDERS:

STUDY COORDINATION CENTRE: Royal Brompton and Harefield NHS Foundation Trust

REC reference:

EudraCT reference:

Sponsor reference:

**Protocol authorised by:**

| **Name & Role** | **Date** | **Signature** |
| --- | --- | --- |
| Dr Nicholas Hopkinson  Principal Investigator | 8rrrr |  |

**Study Management Group**

Chief Investigator: Dr Nicholas Hopkinson (Imperial College)

Co-investigators: Dr Lindsay Edwards (King’s College London)

Dr Alexander Lyon (Imperial College)

Statistician: Dr Winston Banya (Imperial College)

**Study Coordination Centre**

For general queries, supply of trial documentation, and collection of data, please contact:

Study Coordinator: Dr Katrina Curtis

Address: Royal Brompton Hospital, Fulham Rd, London. SW3 6NP

Tel: 020 73518029 E-mail: [k.curtis@imperial.ac.uk](mailto:k.curtis@imperial.ac.uk) Fax: 020 73518939

**Clinical Queries**

Clinical queries should be directed to Dr Katrina Curtis who will direct the query to the appropriate person.

**Sponsor**

Imperial College London is the main research Sponsor for this study. For further information regarding the sponsorship conditions, please contact the Head of Regulatory Compliance at:

Regulatory Compliance

Imperial College London and Imperial College Healthcare NHS Trust

Room 510A

5th floor Lab Block

Charing Cross Hospital

Fulham Palace Road

W6 8RF Tel:  0203 311 0206 Fax: 0203 311 0203

**Funder**

This protocol describes the EDEN-EPIC trial and provides information about procedures for entering participants. The protocol should not be used as a guide for the treatment of other participants; every care was taken in its drafting, but corrections or amendments may be necessary. These will be circulated to investigators in the study, but centres entering participants for the first time are advised to contact the trials centre to confirm they have the most recent version.

Problems relating to this trial should be referred, in the first instance, to the study coordination centre.

This trial will adhere to the principles outlined in the Medicines for Human Use (Clinical Trials) Regulations 2004 (SI 2004/1031), amended regulations (SI 2006/1928) and the International Conference on Harmonisation Good Clinical Practice (ICH GCP) guidelines. It will be conducted in compliance with the protocol, the Data Protection Act and other regulatory requirements as appropriate.

# TABLE OF CONTENTS

| 1 | INTRODUCTION |  |
| --- | --- | --- |
|  |  |  |
| 2 | STUDY OBJECTIVES |  |
|  |  |  |
| 3 | STUDY DESIGN |  |
| 3.1 | STUDY OUTCOME MEASURES |  |
|  |  |  |
| 4 | PARTICIPANT ENTRY |  |
| 4.1 | PRE-RANDOMISATION EVALUATIONS |  |
| 4.2 | INCLUSION CRITERIA |  |
| 4.3 | EXCLUSION CRITERIA |  |
| 4.4 | WITHDRAWAL CRITERIA |  |
|  |  |  |
| 5 | RANDOMISATION AND ENROLMENT PROCEDURE |  |
| 5.1 | RANDOMISATION OR REGISTRATION PRACTICALITIES |  |
| 5.2 | UNBLINDING |  |
|  |  |  |
| 6 | ASSESSMENT AND FOLLOW-UP |  |
| 6.1 | DETAILS OF ASSESSMENTS |  |
| 6.2 | LOSS TO FOLLOW-UP |  |
| 6.3 | TRIAL CLOSURE |  |
|  |  |  |
| 7 | STATISTICS AND DATA ANALYSIS |  |
|  |  |  |
| 8 | MONITORING |  |
| 8.1 | RISK ASSESSMENT |  |
| 8.2 | MONITORING AT STUDY CO-ORDINATION CENTRE |  |
|  |  |  |
| 9 | REGULATORY ISSUES |  |
| 9.1 | CTA |  |
| 9.2 | ETHICS APPROVAL |  |
| 9.3 | CONSENT |  |
| 9.4 | CONFIDENTIALITY |  |
| 9.5 | INDEMNITY |  |
| 9.6 | SPONSOR |  |
| 9.7 | FUNDING |  |
| 9.8 | AUDITS AND INSPECTIONS |  |
|  |  |  |
| 10 | TRIAL MANAGEMENT |  |
|  |  |  |
| 11 | PUBLICATION POLICY |  |
|  |  |  |
| 12 | REFERENCES |  |
|  |  |  |

# Glossary of Abbreviations

| ACE | Angiotensin converting enzyme |
| --- | --- |
| ADP | Adenosine diphosphate |
| ATP | Adenosine triphosphate |
| BR | Beetroot juice |
| CAT | COPD assessment test |
| COPD | Chronic obstructive pulmonary disease |
| CytP450 | Cytochrome P450 |
| FEV_1_ | Forced expiratory volume in one second |
| FFM | Fat free mass |
| FVC | Forced vital capacity |
| GFR | Glomerular filtration rate |
| IC | Inspiratory capacity |
| IHD | Ischaemic heart disease |
| L-NMMA | L-NG-monomethylarginine |
| MRC | Medical Research Council |
| MRS | Magnetic resonance spectroscopy |
| NIRS | Near-infrared spectroscopy |
| NO | Nitric oxide |
| NOS | Nitric oxide synthase |
| PA | Physical activity |
| PAD | Peripheral arterial disease |
| PCr | Phosphocreatine |
| PFT’s | Pulmonary function tests |
| Pi | Inorganic phosphate |
| PL | Placebo |
| PO | Power Output |
| PR | Pulmonary rehabilitation |
| QMVC | Quadriceps maximum voluntary contraction |
| RV | Residual volume |
| SGRQc | St George’s Respiratory Questionnaire for COPD |
| TLC | Total Lung capacity |
| TLco | Transfer factor for carbon monoxide |
| TOI | Tissue oxygenation index |
| TwQ | Twitch quadriceps force |
| VO_2_ | Oxygen uptake |
| XO | Xanthine oxidase |

# Keywords

COPD, nitrate, muscle, exercise

# STUDY SUMMARY

**TITLE** Effect of dietary nitrate supplementation on exercise performance in COPD

**DESIGN** A double blind, placebo-controlled, cross-over study of the effect of nitrate supplementation on time to exhaustion in fixed workload cycle ergometry

**AIMS** To establish whether nitrate supplementation will improve exercise performance in stable COPD patients

**OUTCOME MEASURES** *Primary endpoint* will be time to exhaustion in a fixed workload cycle ergometer test at 70% VO_2max_

*Secondary endpoints* will be

1. Fractional oxygen extraction as assessed by near-infrared spectroscopy (NIRS)
2. Area under the VO_2_ curve to isotime during endurance cycle ergometry

**POPULATION** 25 Stable adult patients with COPD

**ELIGIBILITY** Inclusion -adult patients with COPD (GOLD stage II-IV)

Exclusion -significant comorbidity limiting exercise performance

-significant renal impairment (eGFR<50)

-use of nitrate based medications

-other clinical reason to benefit from nitrates (ischaemic heart disease, peripheral arterial disease)

-within 1 month of pulmonary exacerbation

-within 1 month of pulmonary rehabilitation

**TREATMENT** 140ml of nitrate-rich beetroot juice (0.8g nitrate) vs 140ml of placebo (nitrate-depleted beetroot juice)

**DURATION** 4 weeks

# STUDY OUTLINE


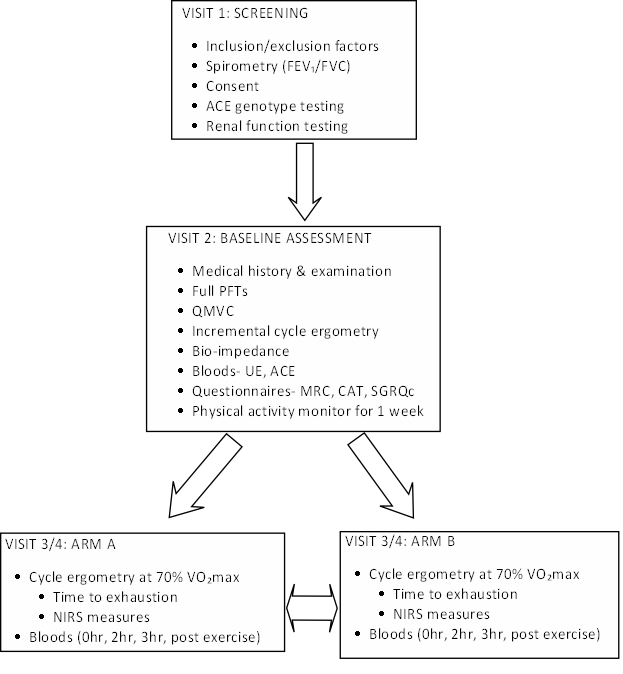


# 1. INTRODUCTION

## BACKGROUND

Chronic obstructive pulmonary disease (COPD) is a disorder characterised by progressive airflow obstruction which is not completely reversible[^1^](#_ENREF_1). The estimated burden of COPD is unfortunately set to rise with the WHO Global Burden of Disease Project predicting in 1997 that it would become the third leading cause of death and fifth leading cause of disability worldwide by 2020[^2^](#_ENREF_2). In fact recent data published in 2013 already ranks COPD as the third leading cause of death worldwide[^3^](#_ENREF_3). The worldwide prevalence of significant disease is estimated to be approximately 10% in those aged over 50 years[^4^](#_ENREF_4).

COPD is a common healthcare concern in the United Kingdom, responsible for considerable mortality and morbidity. Data from primary care services in England suggests significant rates of under-diagnosis when comparison is made to multivariate model-based prevalence estimates, which suggest the true prevalence in England alone is 1.4 million persons[^5^](#_ENREF_5).

Reduced exercise capacity is a major consequence of COPD which had long been attributed to ventilatory limitation and the effect of dyspnoea. However, patients have frequently demonstrated variable exercise capacity despite similar values for airflow obstruction suggesting that loss of pulmonary function is not the sole factor at play in the limitation of exercise capacity. Peripheral muscle strength is itself an important determinant of exercise capacity[^6^](#_ENREF_6) and the sense of muscle fatigue is an important limiting factor in patients with COPD[^7^](#_ENREF_7), often preceding the sense of breathlessness.

COPD is well recognised to have effects beyond pulmonary function, with skeletal muscle dysfunction being a well-recognised and common consequence noted in both early and late disease[^8^](#_ENREF_8). Weakness of the quadriceps, a major muscle group responsible for locomotion, has been demonstrated experimentally via the use of both volitional and non-volitional methods[^9-11^](#_ENREF_9) and is the most commonly studied muscle group in skeletal muscle dysfunction. There is a current unmet need for agents to help augment exercise performance in COPD patients.

## INTRODUCTION TO NITRATE METABOLISM

Nitric oxide (NO) is an important physiological mediator in the body. NO is endogenously produced via the action of the NO synthase (NOS) family of enzymes acting on the amino acid L-arginine, including endothelial NOS (eNOS), neuronal NOS (nNOS) and inducible NOS (iNOS). These enzymes catalyse the conversion of arginine to NO and L-citrulline in a reaction dependent on the presence of molecular oxygen and several cofactors[^12^](#_ENREF_12). Endothelial NOS (eNOS) is itself activated by shear stress on the endothelial membrane, leading to NO production and vasodilatation[^13^](#_ENREF_13)^,^ [^14^](#_ENREF_14).

In addition to the action of the NOS enzyme family, NO is produced via the reduction of exogenous dietary nitrate (NO_3_^-^) in an oxygen independent manner (Figure 1). Nitrate can be found at high levels in leafy greens and beetroot, with vegetables providing 60-80% of our daily dietary intake[^15^](#_ENREF_15). Ingested inorganic nitrate passes readily into the circulation, with the majority being renally excreted[^16^](#_ENREF_16)^,^ [^17^](#_ENREF_17). Approximately 25% of ingested nitrate is actively taken up by the salivary glands via the enterosalivary circulation[^17^](#_ENREF_17), where concentrations may be over 10-fold greater than those measured in the plasma. It is then excreted in saliva and reduced from inert nitrate to bioactive nitrite (NO_2_^-^) via nitrate reductases produced by oral commensal facultative anaerobic bacteria[^18^](#_ENREF_18)^,^ [^19^](#_ENREF_19). Salivary nitrite may be absorbed as saliva is swallowed and enter the circulation directly as nitrite[^18^](#_ENREF_18), or be reduced to nitrogen oxides such as NO in the acidic environment of the stomach[^20-22^](#_ENREF_20) and then absorbed. Thus the supplementation of the diet with nitrate provides a means of increasing circulating plasma nitrite levels which are an indicator of bioavailable NO. The importance of commensal bacteria in facilitating the metabolism of nitrate can be demonstrated by the prevention of the normal rise in plasma nitrite levels after an oral nitrate bolus following the administration of an antibacterial mouthwash[^23^](#_ENREF_23).

The reduction of plasma nitrite to NO is a process enhanced in hypoxic and acidic conditions as may occur in tissues during exercise[^24^](#_ENREF_24)^,^ [^25^](#_ENREF_25). This is an important process as under such conditions hypoxia may be limiting NO generation via the oxygen-dependent NOS pathway[^26^](#_ENREF_26).


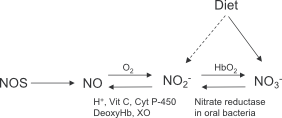


*Figure 1. Nitrate-nitrite-NO pathway in humans. Plasma nitrate originates from the oxidation of endogenously produced NO and dietary sources. Nitrite reduction to NO is enhanced in acidic (H^+^), hypoxic and reducing (e.g. vitamin C) conditions. Other proteins and enzymes may promote this step including Cytochrome P-450 (Cyt P-450), Xanthine Oxidase (XO) and Deoxyhaemoglobin (DeoxyHb). Taken from Lundberg et al.*[*^18^*](#_ENREF_18)

Thus there are two common nitrate-nitrite-NO pathways, via the NOS family of enzymes and via reduction of dietary nitrate. In fact where endogenous NO production is limited via the knock-out of the eNOS gene, the supplementation of nitrite[^27^](#_ENREF_27) or nitrate[^28^](#_ENREF_28) enhances plasma nitrite and nitrate levels towards those seen in normal subjects. This would suggest there is an important interplay between the endogenous and exogenous sources of NO production.

Although health concerns have been raised regarding supplementation with pure sodium nitrate or nitrite due to increased generation of *N*-nitrosamines with increased risk of gastric cancer, the generation of nitric oxides from nitrite is thought to be an important physiological pathway in humans. In fact it has been proposed that nitrite is an important source of reactive nitrogen intermediates including NO that possess important antimicrobial activity, gastric protective effects and control of vascular tone particularly in ischaemic tissues[^21^](#_ENREF_21). In addition nitrate-rich whole vegetables or juices also contain antioxidants and polyphenols that may reduce the formation of harmful nitrogenous compounds[^29^](#_ENREF_29)^,^ [^30^](#_ENREF_30). Thus the general consensus is that nitrate supplementation from vegetable sources is likely to be an important component of a healthy diet.

Abnormalities in NO synthesis occur in ageing and may be linked to poor exercise tolerance seen in old age. Older subjects show reduced plasma nitrite production in response to exercise than younger individuals [^31^](#_ENREF_31). As nitrite is the main oxidation product of NO this is reflective of reduced eNOS activity during exertion, and as such can be abolished by the systemic administration of the NOS inhibitor L-NMMA (L-NG-monomethylarginine). This reduced NOS activity is accompanied by impaired endothelial vasodilator function in response to exercise in older persons as assessed by brachial artery flow mediated dilation studies[^31^](#_ENREF_31).

## NITRATE SUPPLEMENTATION AND EXERCISE PHYSIOLOGY

A link between plasma nitrite levels and exercise performance has been recognised suggesting a role for the nitrate-nitrite-NO pathway in augmenting exercise. Rassaf et al. (1997)[^32^](#_ENREF_32) noted that in 55 healthy subjects post-exercise nitrite plasma levels correlated with exercise performance in an ergometric exercise test, and Dreißigacker et al. (2010)[^33^](#_ENREF_33) documented a correlation between plasma nitrite levels and performed work in a group of 22 healthy male subjects performing cycle ergometry. As nitrate supplementation leads to a rise in both plasma nitrate and nitrite levels there has thus been much interest in the effect of nitrate supplementation on exercise performance, either through supplementation of pure sodium nitrate[^34-36^](#_ENREF_34), beetroot juice rich in nitrate[^24^](#_ENREF_24)^,^ [^37-42^](#_ENREF_37) or whole beetroot itself[^43^](#_ENREF_43).

Research predominantly from the field of sports medicine has demonstrated a reduction in the oxygen cost to perform a submaximal workload via a variety of exercise modalities following nitrate supplementation in healthy individuals[^24^](#_ENREF_24)^,^ [^36-39^](#_ENREF_36) as indicated by reduced steady state VO_2_ amplitude for a given low or moderate-intensity work rate (Figure 2). This has in turn correlated with improved measures of exercise performance[^24^](#_ENREF_24)^,^ [^37-39^](#_ENREF_37)^,^ [^43^](#_ENREF_43). Lansley et al. (2011)[^38^](#_ENREF_38) compared dietary supplementation with beetroot juice versus placebo in a randomised cross-over design in 9 healthy, physically active males who undertook walking and moderate and severe-intensity running exercise. Beetroot juice reduced the oxygen cost of all exercise modalities and extended the time to exhaustion during severe-intensity running by 15% (from 7.6 to 8.7 minutes). This is an impressive finding as the oxygen cost of exercise is known to be uninfluenced by such interventions as prior exercise[^44^](#_ENREF_44) and oxygen supplementation[^45^](#_ENREF_45).


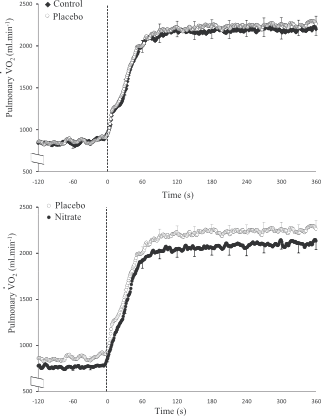


*Figure 2: Pulmonary uptake (VO2) responses during step increment to a moderate intensity running speed from walking pace (represented by dashed line). There was no significant change comparing group mean responses for non-supplemented control and placebo supplementation (upper panel). There was a significant reduction in the group mean VO_2_ when administered nitrate-rich beetroot juice supplementation versus placebo (lower panel). Taken from Lansley et al. 2011*[*^38^*](#_ENREF_38)*.*

Similar effects have been demonstrated when exercising at a high intensity constant workload. Studies have shown reduction in whole body oxygen uptake (VO_2max_) amplitude in combined arm and leg exercise[^34^](#_ENREF_34), cycling[^35^](#_ENREF_35), repetitive leg extension[^24^](#_ENREF_24) and running[^38^](#_ENREF_38) at high intensity, indicating improved exercise efficiency and reduced oxygen cost. Two further studies have shown extended time-to exhaustion[^37^](#_ENREF_37), improved time-trial performance[^40^](#_ENREF_40) and improved power output (PO):VO_2_ ratio[^40^](#_ENREF_40) indicating improved overall exercise performance and economy when exercising at high intensity.

Reduction in the oxygen cost of exercise has not, however, always been reproducible in experiments testing highly trained or elite athletes[^35^](#_ENREF_35)^,^ [^41^](#_ENREF_41)^,^ [^42^](#_ENREF_42). Wilkerson et al. (2012)[^41^](#_ENREF_41) who enrolled 8 well trained club-level cyclists show no significant change to the power output during a laboratory based 50 mile time trial although there was a non-significant tendency for the VO_2_ to lower, thus leading to a significantly greater PO:VO_2_ ratio in the group receiving nitrate supplementation. This did not, however, correlate with an improved performance in a time trial setting. The increase in plasma nitrite levels seen in the group was, however, lower following supplementation than that previously observed, in part because of higher baseline nitrite and nitrate levels as has previously been seen in athletic subjects[^35^](#_ENREF_35)^,^ [^46^](#_ENREF_46)^,^ [^47^](#_ENREF_47). The mean baseline plasma nitrite level of the subjects in the study by Wilkerson et al. (2012)[^41^](#_ENREF_41) was recorded as 389 ± 107 nM as compared to the study by Lansley et al. (2011)[^38^](#_ENREF_38) using healthy but non-trained persons who had a baseline plasma nitrite level of 197 ± 184 nM. The administration of 500 ml of nitrate-rich beetroot juice (containing 6.2mmol nitrate) increased plasma nitrite levels by 105% in the untrained group (VO_2max_ 55 ± 7 ml.kg^-1^.min^-1^)[^38^](#_ENREF_38) as opposed to only 21% in the well-trained athletes (VO_2max_ 63 ± 8 ml.kg^-1^.min^-1^)[^41^](#_ENREF_41).

The failure to show the same amplitude of response in trained individuals may thus be attributed in part to pre-existing training induced upregulation of NOS and greater activation of the nitrate-nitrite-NO pathway[^48^](#_ENREF_48)^,^ [^49^](#_ENREF_49). Thus well trained athletes having high endogenous levels of NO and an already efficient oxidative metabolism may not respond to supplementation of further nitrate via dietary means or may require higher dosages to show a significant effect. Additionally such individuals are likely to choose a diet high in natural nitrate and the study by Wilkerson et al.[^41^](#_ENREF_41) did not modulate normal dietary intake during the period of observation. However, some further evidence has been contradictory. The study by Christensen et al.[^42^](#_ENREF_42) also failed to show a response in highly trained cyclists (VO_2max_ 72 ± 4 ml.kg^-1^.min^-1^) as a whole, however in contrast to that seen in previous studies a subgroup comparison of ‘responders’ versus ‘non-responders’ showed no difference in the baseline nitrate levels (a surrogate measure of nitrite levels) indicating that probably several factors are at play and endogenous NO production is not the sole influence. It is also feasible that trained individuals have already established improved whole body efficiency, meaning that there is less available room for further improvement in aerobic exercise performance and thus endurance capacity through nutritional interventions[^50^](#_ENREF_50).

An interesting study in 12 well-trained apnoea divers by Engan et al. (2012)[^51^](#_ENREF_51) demonstrated acute nitrate supplementation increased maximal resting apnoea duration by 11%[^51^](#_ENREF_51). This suggests that the reduced metabolic cost mediated by nitrate supplementation may occur even in a non-exercising state.

Studies using beetroot juice specifically depleted of nitrate by passage through an ion exchange resin prior to pasteurisation developed by the University of Exeter have shown the effects are dependent in large part on high levels of nitrate, and can thus not be attributed to other organic substances present in beetroot[^38^](#_ENREF_38), including betaine, polyphenols and antioxidants. Of course a synergistic effect of nitrate with these and other organic compounds cannot be excluded by the use of such methods.

Administration of nitrate rich foodstuffs such as beetroot juice has been demonstrated to exert effects at 2 to 3 hours post administration, coincident with the rise in plasma nitrite levels[^39^](#_ENREF_39)^,^ [^40^](#_ENREF_40)^,^ [^52^](#_ENREF_52) indicating acute effects may be seen even after the administration of a single bolus dose. The effect of daily administration on exercise performance has been demonstrated to continue up to 15 days with significant increases in VO_2max_ and power output[^39^](#_ENREF_39) shown. Thus up to this time point there has been no demonstration of tolerance to the effect of nitrate supplementation.

## PROPOSED MECHANISMS OF NITRATE ACTION

There are several potential mechanisms by which the administration of nitrate may reduce the oxygen cost of submaximal exercise. These include increased steady-state utilisation of anaerobic pathways, augmented contractile efficiency with reduced ATP turnover to perform a set amount of work and reduced oxygen cost of ATP resynthesis by increasing the mitochondrial P/O ratio. The P/O ratio is measured in terms of the amount of oxygen consumed per molecule of ATP produced (a higher ratio indicating more efficient oxidative phosphorylation[^53^](#_ENREF_53)). ATP supply to muscle is via three main sources: glycolysis, ATP regeneration from phosphocreatine and oxidative phosphorylation (Figure 3).


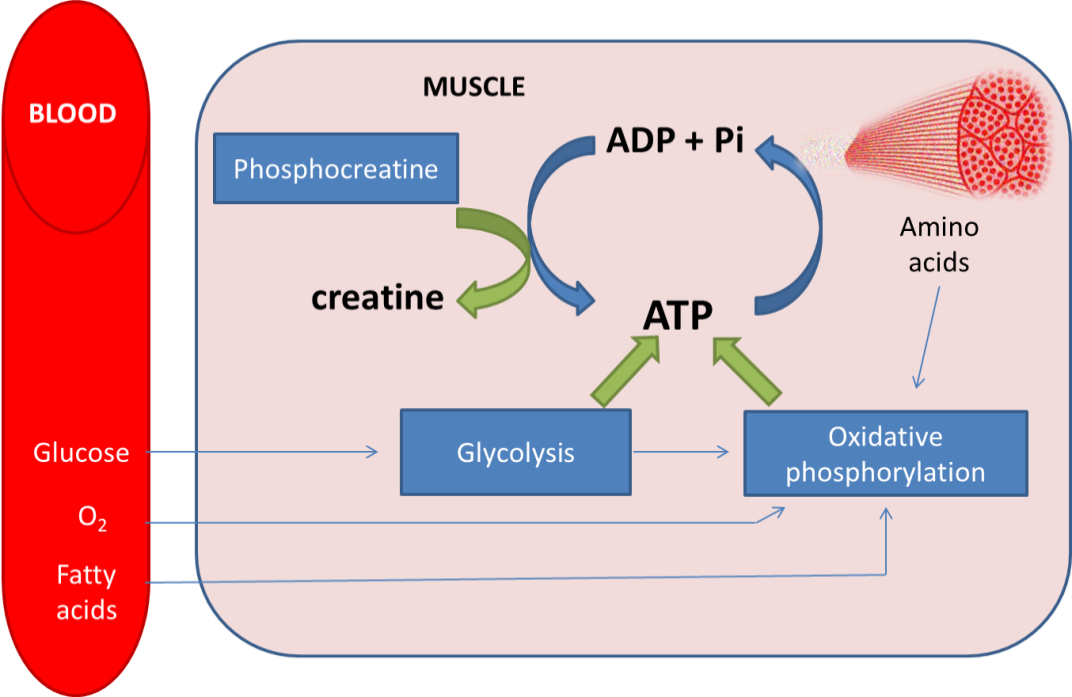


*Figure 3: Schematic diagram illustrating sources of ATP in muscle.*

Several experimental methods have used serum lactate as a surrogate measure of anaerobic metabolism[^36^](#_ENREF_36)^,^ [^37^](#_ENREF_37), demonstrating a failure of alteration in blood lactate levels during exercise following nitrate administration as compared to placebo. This is a highly simplified and limited approach, and although suggests anaerobic pathways are not increasingly favoured following nitrate administration, does not effectively exclude that this may be contributing to its mode of action.

Investigators using phosphorus-31 magnetic resonance spectroscopy (^31^P-MRS) have suggested that at least in part nitrate supplementation acts via reduced ATP cost of muscle force production[^24^](#_ENREF_24). This experimental method provides information on tissue [phosphocreatine] ([PCr]) degradation and adenosine diphosphate [ADP] and inorganic phosphate [Pi] accumulation during exercise, along with pH measures and pulmonary VO_2_ which provides an estimate of skeletal muscle O_2_ consumption. Thus estimates can be made of the total ATP turnover rate and the metabolic changes that occur during exercise, as well as the relative contributions of phosphocreatine hydrolysis, glycolysis and oxidative phosphorylation to ATP regeneration during exercise. Measures obtained by ^31^P-MRS provide an excellent surrogate of events at a cellular level and an insight into in vivo muscle cell function.

Bailey et al. (2010)[^24^](#_ENREF_24) demonstrated that nitrate supplementation leads to reduced overall ATP turnover rate, with reduced ADP and Pi accumulation, and reduced relative contributions to ATP regeneration from phosphocreatine degradation and oxidative phosphorylation. Whilst maintaining the same power output the ATP requirement was reduced[^24^](#_ENREF_24), implying an improved muscle efficiency and reduced tendency to fatigue by improved coupling between ATP hydrolysis and muscle force generation. There was no relative increased contribution from anaerobic glycolysis suggesting that the reduced ATP requirement was not dependent on increased utilisation of anaerobic metabolic pathways[^24^](#_ENREF_24). The finding are consistent with known control factors for oxidative phosphorylation given that ATP resynthesis is itself stimulated by ATP usage and products of ATP hydrolysis[^54^](#_ENREF_54)^,^ [^55^](#_ENREF_55).

Lansley et al. (2011)[^38^](#_ENREF_38) showed no alteration in the recovery of phosphocreatine following knee extensor exercise or muscle maximum oxidative capacity (Qmax) in the treatment group receiving nitrate supplementation as compared to placebo. As a surrogate measure of oxidative ATP synthesis they suggested these results were an indicator that nitrate was not acting via enhanced ATP synthesis. Of note, however, unless oxygen delivery is a limiting factor, improved thermodynamic efficiency may be achieved even in the absence of an increase in maximal mitochondrial function.

It is important to consider how nitrate metabolites may be able to reduce overall ATP turnover in the contracting myocyte. The generation of ATP by oxidative phosphorylation is dependent on generation and maintenance of the electrochemical proton gradient. However, not all of this membrane potential is eventually dissipated in the production of ATP as there is not 100% coupling to ATP production due to proton leakage across the inner mitochondrial membrane and through uncoupling proteins.

Larsen et al. (2011)[^56^](#_ENREF_56) demonstrated in vitro that in harvested human skeletal muscle mitochondria 3 days of nitrate supplementation increased oxidative phosphorylation efficiency, the P/O ratio showing an increase of 19% and ATP production increasing by 23%. This correlated with a significant 3% reduction in whole-body oxygen consumption during submaximal workload exercise in the same healthy subjects from which the skeletal muscle biopsies had been obtained[^56^](#_ENREF_56). Thus nitrate supplementation may both improve the efficiency of muscle contraction (via as yet unknown mechanisms) and of ATP generation via oxidative phosphorylation. Enhanced efficiency of oxidative phosphorylation may be through the known reversible inhibition of cytochrome oxidase by NO with reduced proton slippage[^57^](#_ENREF_57) as has been demonstrated in in vitro studies of mitochondrial function in isolated rat liver mitochondria. In fact mitochondrial NOS is located in close proximity to cytochrome oxidase and deletion of the domain that anchors NOS in the mitochondrial outer membrane leads to increased oxygen consumption[^58^](#_ENREF_58), providing further support that NO may be a physical regulator of this enzyme. NO may also improve coupling of oxidation to ATP production by reduction in proton leak and slippage[^57^](#_ENREF_57) and thus maintenance of the electrochemical proton gradient responsible for ATP synthesis.

NO may also be acting at the level of ATPase dependent pathways in skeletal muscle making them more efficient, the most ATP consuming being the actin-myosin ATPase crucial for cross-bridge cycling, and calcium transport across the sarcoplasmic reticulum[^59^](#_ENREF_59). Reducing the ATP cost of these energy dependent process leads to reduced overall ATP turnover and oxygen cost of muscle contraction[^60^](#_ENREF_60)^,^ [^61^](#_ENREF_61). Physiological concentrations of NO have been demonstrated to alter striated muscle myosin cross-bridge kinetics from a high-speed low-force state, to a low-speed high-force generating state in an in vitro preparation[^62^](#_ENREF_62). NO is also known to influence calcium handling by sarcoplasmic reticulum (Viner et al. 2002) and in a rabbit femoral striated muscle preparation NO has also been shown to reduce sarcoplasmic reticululm Ca-ATPase (SERCA) activity[^63^](#_ENREF_63).

NO is a known activator of guanylate cyclase which synthesises cyclic guanosine monophosphate (cGMP) from guanosine triphosphate (cGTP) leading to the relaxation of vascular smooth muscle. Thus not surprisingly the administration of NO_3_^-^ has been correlated with reduced systolic and diastolic blood pressure[^52^](#_ENREF_52)^,^ [^64^](#_ENREF_64) and potential cardioprotective effects. As NO is produced more readily in hypoxic and acidotic conditions this provides one mechanism of action of NO, by improving blood flow to metabolically active skeletal muscle ensuring more efficient matching of oxygen supply to local metabolic demand. By being able to meet the metabolic demands of exercising tissue this may permit more prolonged exercise.

Available data on nitrate supplementation on exercise performance so far extends up to 15 days of supplementation. Although Larsen et al. (2011)[^56^](#_ENREF_56) showed no alteration in mitochondrial biogenesis or density following nitrate supplementation up to 3 days, this is certainly not to say that such mechanisms may not be influential following more prolonged periods of supplementation which have yet to be studied. Thus it is plausible that nitrate may affect total mitochondrial number as well as individual mitochondrial activity, although this has yet to be proven.

## ROLE OF NITRATE IN HYPOXIC CONDITIONS

NO may also be an important mediator in the adaptation to life in a hypoxic environment. Observational studies of high altitude dwelling humans have shown higher levels of exhaled NO[^65^](#_ENREF_65) and plasma nitrite[^66^](#_ENREF_66) than lowlanders. This cannot be accounted for by increased dietary consumption of nitrate as the average daily consumption studied would have been insufficient to increase plasma levels of nitrate and nitrite significantly[^66^](#_ENREF_66). This suggests such persons adapted to life at altitude have upregulation of endogenous NO that may help them meet this physiological challenge.

In line with studies from sports medicine, comparisons between long-term Tibetan residents and newly acclimatised residents at altitude show lower VO_2max_ in the Tibetan group and higher workload at maximal exercise effort illustrating possible improved exercise efficiency of the Tibetan population[^67^](#_ENREF_67). This suggests a possible causal mechanism related to upregulation of endogenous NO.

Further to this in studies of high altitude sickness, there is a noted association with reduced exhaled NO[^68^](#_ENREF_68) and polymorphisms of endothelial NOS[^69^](#_ENREF_69). This may indicate an adaptive role for NO in the physiological challenge of the hypoxia of altitude by reducing the oxygen cost of physical activity. In support, enhancing dietary nitrate has been demonstrated to reduce the oxygen cost of exercise at a submaximal workload in an hypoxic environment[^70^](#_ENREF_70)^,^ [^71^](#_ENREF_71). Masschelein et al. (2012)[^70^](#_ENREF_70) tested 15 healthy volunteers via submaximal workload cycle ergometry at both normoxia and in hypoxic conditions (11% oxygen) after loading with nitrate supplementation or placebo control. VO_2_ was notably lower at both rest and during submaximal exercise after nitrate supplementation than control, and was associated with improved tissue oxygenation index and lower rate of muscle oxygen extraction as assessed by near infrared spectroscopy[^70^](#_ENREF_70). This effect seems to be independent of increased muscle blood flow using indirect measures via NIRS, suggesting that upregulation of NO has effects beyond simply that on the vasculature[^70^](#_ENREF_70). Nitrate supplementation has also been shown to attenuate the perturbation in muscle metabolism seen in hypoxia. Vanhatalo et al. (2011) tested 9 healthy subjects via incremental knee extension exercise to the limit of tolerance, showing prior treatment with nitrate significantly increased the exercise time and improved the kinetics of phosphocreatine recovery post exercise.

These studies are clearly of interest in the study of chronic respiratory disease such as COPD, which is frequently associated with tissue hypoxia and ventilatory limitation. In those with chronic lung disease simply performing the activities of daily living requires energy that represents a high proportion of VO_2max_, thus any intervention that reduces the oxygen cost in conditions where oxygen supply is limited has the potential to improve functional abilities and quality of life in this patient population. Improving skeletal muscle blood flow and the efficiency of energy metabolism in working tissues has significant potential to improve physical activity, and potentially via a cost-effective simple nutritional intervention.

Pre-existing clinical studies have been undertaken in patients with peripheral arterial disease where oxygen delivery is unable to meet the demands of metabolically active tissue of the legs during walking; leading to the development of claudicant pain that then limits further exertion. Kenjale et al. (2011)[^72^](#_ENREF_72) studied 9 patients with peripheral vascular disease in an open-label, randomised, cross-over trial of nitrate supplementation versus placebo. The supplementation of nitrate by beetroot juice administration has been shown to improve both walking distance and time before the onset of claudicant pain, by 18% and 17% respectively, in an incremental walking test[^72^](#_ENREF_72). This is associated with lower gastrocnemius fractional oxygen extraction as assessed by near infrared spectroscopy[^72^](#_ENREF_72) indicating improved energy efficiency.

## APPLICATION OF NEAR INFRARED SPECTROSCOPY (NIRS)

NIRS provides a semi-quantitative non-invasive measure of tissue oxygenation status[^73^](#_ENREF_73). NIR light penetrates biological tissues and allows the detection of changes in light-absorbing molecules such as haemoglobin in humans. The intensity of incident and transmitted light is used to estimate concentration changes from the resting baseline for oxygenated (HbO_2_), deoxygenated (HHb) and total tissue haemoglobin/myoglobin (THb) in the microvasculature of the muscle.

Changes in tissue oxygenation status can be measured to estimate the fractional oxygen extraction. The HHb signal is considered to be a surrogate of microvascular oxygen extraction as it is little influenced by changes in blood volume[^74^](#_ENREF_74), thus its amplitude can provide a non-invasive index of fractional oxygen extraction. The ratio of oxyhaemoglobin to total haemoglobin can also be studied to allow measurement of the tissue oxygenation index (TOI). Studies have demonstrated reduced fractional oxygen extraction as assessed by NIRS during exercise in both healthy adults receiving nitrate supplementation[^37^](#_ENREF_37) and in adults diagnosed with peripheral arterial disease[^72^](#_ENREF_72).

# 2. STUDY OBJECTIVES

The purpose of this study is to investigate the effects of an acute administration of beetroot (BR) juice versus placebo beverage ingestion on plasma NO_2_^-^ levels, blood pressure, exercise tolerance and fractional oxygen extraction.

The following hypotheses are to be tested:

1) BR would increase plasma NO_2_^-^ levels (a biomarker of NO production and availability)

2) BR would increase cycling time to exhaustion at a fixed workrate (70% peak)

3) BR will reduce oxygen consumption during equivalent exercise (area under VO_2_ curve to isotime)

4) BR would reduce fractional oxygen extraction of the quadriceps as assessed by NIRS

# 3. STUDY DESIGN

The study is a randomised, double-blind, cross-over, placebo-controlled trial. 25 patients with COPD will be randomised to receive nitrate-rich beetroot juice or placebo as a bolus dose 3 hours prior to endurance cycle ergometry, and then cross-over to the other intervention.

### 3.1 STUDY OUTCOME MEASURES

*Primary endpoint* will be an increase in time to exhaustion at 70% peak workload on a cycle ergometer.

*Secondary endpoints* will be:

1. Area under VO_2_ curve to isotime (VO_2_) during endurance cycle ergometry;
2. Fractional oxygen extraction of quadriceps muscle as assessed by NIRS.
3. Mitochondrial DNA levels
4. Branched chain amino acid levels

# 4. PARTICIPANT ENTRY

### 4.1 PRE-RANDOMISATION EVALUATIONS

Patients will have a clinical and spirometric diagnosis of COPD.

### 4.2 INCLUSION CRITERIA

Patients will need to have a clinical and spirometric diagnosis of COPD, GOLD stage II-IV.

### 4.2 EXCLUSION CRITERIA

Clinically unstable patients (within one month of exacerbation), significant comorbidity limiting exercise tolerance, significant renal impairment (estimated glomerular filtration rate (eGFR) <50 ml.min^-1^), hypotension (systolic blood pressure <100 mmHg), pregnancy, use of nitrate based medication, other reason for benefit from nitrate supplementation (ischaemic heart disease, peripheral arterial disease), use of long-term oxygen therapy.

### 4.3 WITHDRAWAL CRITERIA

Patients will be withdrawn from the study if: they have a significant adverse event or if they withdraw their consent for participation. If patients withdraw their consent their data will be destroyed.

# 5. RANDOMISATION AND ENROLMENT PROCEDURE

## 5.1 RANDOMISATION OR REGISTRATION PRACTICALITIES

Patients will be randomly assigned to the order in which they receive nitrate-rich beetroot juice versus placebo. Randomisation will be performed using block randomisation with consecutive numbers linked to identical preparations of placebo or active treatment. The list will be generated by Winston Banya, Trust statistician.

## 5.2 UNBLINDING

If necessary this will be done through Winston Banya who will hold an unblinded list.

# 6. ASSESSMENT AND FOLLOW-UP

See appendix for table.

Patients with diagnosed COPD will be approached to consider participation in the study either in clinic or through the department’s research audit database.

Visit 1 -Inclusion/exclusion criteria reviewed including spirometry.

-Consent taken.

-Blood sample for renal function (if not tested in the previous 12 months).

Visit 2 -Baseline medical history and clinical examination including blood pressure, anthropometrics and measurement of body composition.

-Blood withdrawal for analysis.

-Full pulmonary function testing if not performed within 3 months.

-Quadriceps maximal voluntary contraction.

-Activity monitor supplied to wear for one week.

-Questionnaires.

-Incremental cycle ergometry for determination of peak workload and VO_2max_.

Visit 3/4 -Fixed rate, symptom limited cycle ergometry (at 70% peak workload VO_2max_) after dosing with beetroot juice (BR) or placebo (PL) - time to exhaustion measured VO_2_ and fractional oxygen extraction as assessed by NIRS.

-Blood withdrawal at times 0hr (arrival), 3hr (pre exercise), at peak exercise and 15 minutes post exercise (with BR or PL being administered at 0hr).

Subjects will be asked to avoid nitrate-rich foods in the 48hr prior to the day of testing for visits 3 and 4, and to avoid any strenuous exertion in the 24hr preceding period. They will be asked to avoid cooked food for breakfast and eat the same meal prior to both visits 3 and 4 and ensure that caffeine consumption is matched. They will also be asked to avoid use of any mouthwash or chewing gum which removes the oral bacteria responsible for nitrate reductase activity[^23^](#_ENREF_23).

Testing for visits 3 and 4 should occur at the same time of day (+/- 2hr). Subjects will be given a 140ml volume of BR or PL to consume at time 0hr. This volume of BR provides 0.8g nitrate (12.9 mmol).

There is a minimum 7 day washout period between visits 3 and 4.

## 6.1 DETAILS OF ASSESSMENTS

-Blood samples: At visit 1 or 2 patients will have a baseline measurement of their renal function. At visits 3 and 4 serial blood samples will be taken to measure plasma nitrite levels and to measure mitochondrial DNA and branched chain amino acids (a marker of muscle damage)

- Mouth nitrate levels –mouth washings will be frozen and stored for subsequent analysis to measure nitrate levels in the mouth (due to commensal bacteria)

-Fat free mass: Will be determined using bioelectrical impedance analysis by measuring the electrical resistance between the wrist and ankle using a Bodystat 1500 device. This value is dependent on body water content and based on this fat free mass can be calculated using a disease specific regression equation[^75^](#_ENREF_75).

-Lung function: Will be measured in the lung function department of the Royal Brompton Hospital according to international guidelines and with rigorous quality assurance in place with a Jaeger master lab system. Spirometry, gas transfer and plethysmographic lung volumes (TLC, RV, FRC) will also be measured. All lung function tests will be performed with patients taking their usual regular medication.

-Quadriceps strength: Maximum isometric quadriceps force (QMVC) will be measured using the technique of Edwards et al.[^76^](#_ENREF_76) The maximum force generated during at least three maximum voluntary contractions with vigorous encouragement is measured.

- Incremental Cycle ergometry: A symptom limited incremental exercise test will be performed on a cycle ergometer with metabolic measurements collected using a mouthpiece. Following a 2 minute rest period and 2 minutes free cycling workload will increase by 5 Watts every 30 seconds. Measurements will include peak workload, VO_2_, VCO_2_, minute ventilation, respiratory rate and tidal volume.

-Endurance cycle ergometry test at a fixed workload (70% peak workload). Two minutes at rest, 2 minutes free cycle, then at a fixed workload (70% peak watts) until exhaustion. Standardised encouragement will be given and patients will be asked about limiting symptoms.

-Physical activity monitoring: Patients will wear a multiaxial physical activity monitor continually for a week as part of their baseline assessment. This will record step count and physical activity level. The monitor can be returned by post.

## 6.2 LOSS TO FOLLOW-UP

Efforts will be made to contact patients by post and telephone and be reference to their GPs. Where it is not possible to contact them their vital status will be reviewed by referring to the ONS database 3 months after the planned end of their study participation.

## 6.3 TRIAL CLOSURE

The end of the trial will be defined as when the last patient has attended for their last visit.

# 7. STATISTICS AND DATA ANALYSIS

The primary endpoint will be the change in time to exhaustion in a fixed workload cycle ergometer test. This is a validated endpoint in COPD, having been previously in studies of therapeutic interventions[^77^](#_ENREF_77).

Data and all appropriate documentation will be stored for a minimum of 10 years after the completion of the study, including the follow-up period.

As this is a pilot study a pragmatic sample size of 25 has been selected as offering a good chance of identifying a clinically meaningful effect.

# 8. MONITORING

## 8.1 RISK ASSESSMENT

The study is considered to be low risk. The assessments are routine in clinical practice and the intervention involves a nutritional product.

# 9. REGULATORY ISSUES

## 9.1 ETHICS APPROVAL

The Study Coordination Centre has obtained approval from the Bromley Research Ethics Committee. The study must be submitted for Site Specific Assessment (SSA) at each participating NHS Trust. The Study Coordination Centre will require a copy of the Trust R&D approval letter before accepting participants into the study. The study will be conducted in accordance with the recommendations for physicians involved in research on human subjects adopted by the 18th World Medical Assembly, Helsinki 1964 and later revisions.

## 9.2 CONSENT

Consent to enter the study must be sought from each participant only after a full explanation has been given, an information leaflet offered and time allowed for consideration. Signed participant consent should be obtained. The right of the participant to refuse to participate without giving reasons must be respected. After the participant has entered the trial the clinician remains free to give alternative treatment to that specified in the protocol at any stage if he/she feels it is in the participant’s best interest, but the reasons for doing so should be recorded. In these cases the participants remain within the study for the purposes of follow-up and data analysis. All participants are free to withdraw at any time from the protocol treatment without giving reasons and without prejudicing further treatment.

## 9.3 CONFIDENTIALITY

Participants’ identification data will be required for the registration process. The Study Coordination Centre will preserve the confidentiality of participants taking part in the study and is registered under the Data Protection Act.

## 9.4 INDEMNITY

Imperial College London holds negligent harm and non-negligent harm insurance policies which apply to this study.

## 9.5 SPONSOR

Imperial College London will act as the main Sponsor for this study. Delegated responsibilities will be assigned to the NHS trusts taking part in this study.

## 9.6 FUNDING

The study is funded from departmental funds.

## 9.7 AUDITS AND INSPECTIONS

The study may be subject to inspection and audit by Imperial College London under their remit as Sponsor, the Study Coordination Centre and other regulatory bodies to ensure adherence to GCP.

# 10. TRIAL MANAGEMENT

A Trial Management Group (TMG) will be appointed and will be responsible for overseeing the progress of the trial. The day-to-day management of the trial will be co-ordinated through the Royal Brompton Study Coordination Centre.

# 11. PUBLICATION POLICY

All publications and presentations relating to the study will be authorised by the Trial Management Group. The first publication of the trial results will be in the name of the Trial Management Group, if this does not conflict with the journal’s policy. If there are named authors, these will include at least the trial’s Chief Investigator, Statistician and Trial Coordinator. Members of the TMG and the Data Monitoring Committee will be listed and contributors will be cited by name if published in a journal where this does not conflict with the journal’s policy. Authorship of parallel studies initiated outside of the Trial Management Group will be according to the individuals involved in the project but must acknowledge the contribution of the Trial Management Group and the Study Coordination Centre.

# 12. REFERENCES

1. Devereux, G., *ABC of chronic obstructive pulmonary disease. Definition, epidemiology, and risk factors.* BMJ, 2006. **332**(7550): p. 1142-4.

2. Murray, C.J. and A.D. Lopez, *Alternative projections of mortality and disability by cause 1990-2020: Global Burden of Disease Study.* Lancet, 1997. **349**(9064): p. 1498-504.

3. Lozano, R., et al., *Global and regional mortality from 235 causes of death for 20 age groups in 1990 and 2010: a systematic analysis for the Global Burden of Disease Study 2010.* Lancet, 2013. **380**(9859): p. 2095-128.

4. Buist, A.S., et al., *International variation in the prevalence of COPD (the BOLD Study): a population-based prevalence study.* Lancet, 2007. **370**(9589): p. 741-50.

5. Nacul, L., et al., *COPD in England: a comparison of expected, model-based prevalence and observed prevalence from general practice data.* J Public Health (Oxf), 2011. **33**(1): p. 108-16.

6. Gosselink, R., T. Troosters, and M. Decramer, *Peripheral muscle weakness contributes to exercise limitation in COPD.* Am J Respir Crit Care Med, 1996. **153**(3): p. 976-80.

7. Killian, K.J., et al., *Exercise capacity and ventilatory, circulatory, and symptom limitation in patients with chronic airflow limitation.* Am Rev Respir Dis, 1992. **146**(4): p. 935-40.

8. Seymour, J.M., et al., *The prevalence of quadriceps weakness in COPD and the relationship with disease severity.* Eur Respir J, 2010. **36**(1): p. 81-8.

9. Bernard, S., et al., *Peripheral muscle weakness in patients with chronic obstructive pulmonary disease.* Am J Respir Crit Care Med, 1998. **158**(2): p. 629-34.

10. Man, W.D., et al., *Non-volitional assessment of skeletal muscle strength in patients with chronic obstructive pulmonary disease.* Thorax, 2003. **58**(8): p. 665-9.

11. Man, W.D., et al., *Abdominal muscle and quadriceps strength in chronic obstructive pulmonary disease.* Thorax, 2005. **60**(9): p. 718-22.

12. Alderton, W.K., C.E. Cooper, and R.G. Knowles, *Nitric oxide synthases: structure, function and inhibition.* Biochem J, 2001. **357**(Pt 3): p. 593-615.

13. Furchgott, R.F. and J.V. Zawadzki, *The obligatory role of endothelial cells in the relaxation of arterial smooth muscle by acetylcholine.* Nature, 1980. **288**(5789): p. 373-6.

14. Ignarro, L.J., et al., *Endothelium-derived relaxing factor produced and released from artery and vein is nitric oxide.* Proc Natl Acad Sci U S A, 1987. **84**(24): p. 9265-9.

15. Ysart, G., et al., *Dietary exposures to nitrate in the UK.* Food Addit Contam, 1999. **16**(12): p. 521-32.

16. Lundberg, J.O. and E. Weitzberg, *NO generation from inorganic nitrate and nitrite: Role in physiology, nutrition and therapeutics.* Arch Pharm Res, 2009. **32**(8): p. 1119-26.

17. Lundberg, J.O. and E. Weitzberg, *NO-synthase independent NO generation in mammals.* Biochem Biophys Res Commun, 2010. **396**(1): p. 39-45.

18. Lundberg, J.O. and M. Govoni, *Inorganic nitrate is a possible source for systemic generation of nitric oxide.* Free Radic Biol Med, 2004. **37**(3): p. 395-400.

19. Duncan, C., et al., *Chemical generation of nitric oxide in the mouth from the enterosalivary circulation of dietary nitrate.* Nat Med, 1995. **1**(6): p. 546-51.

20. Benjamin, N., et al., *Stomach NO synthesis.* Nature, 1994. **368**(6471): p. 502.

21. Lundberg, J.O., et al., *Nitrate, bacteria and human health.* Nat Rev Microbiol, 2004. **2**(7): p. 593-602.

22. Lundberg, J.O., et al., *Intragastric nitric oxide production in humans: measurements in expelled air.* Gut, 1994. **35**(11): p. 1543-6.

23. Govoni, M., et al., *The increase in plasma nitrite after a dietary nitrate load is markedly attenuated by an antibacterial mouthwash.* Nitric Oxide, 2008. **19**(4): p. 333-7.

24. Bailey, S.J., et al., *Dietary nitrate supplementation enhances muscle contractile efficiency during knee-extensor exercise in humans.* J Appl Physiol, 2010. **109**(1): p. 135-48.

25. Richardson, R.S., et al., *Cellular PO2 as a determinant of maximal mitochondrial O(2) consumption in trained human skeletal muscle.* J Appl Physiol, 1999. **87**(1): p. 325-31.

26. Stuehr, D.J., et al., *Update on mechanism and catalytic regulation in the NO synthases.* J Biol Chem, 2004. **279**(35): p. 36167-70.

27. Bryan, N.S., et al., *Dietary nitrite restores NO homeostasis and is cardioprotective in endothelial nitric oxide synthase-deficient mice.* Free Radic Biol Med, 2008. **45**(4): p. 468-74.

28. Carlstrom, M., et al., *Dietary inorganic nitrate reverses features of metabolic syndrome in endothelial nitric oxide synthase-deficient mice.* Proc Natl Acad Sci U S A, 2010. **107**(41): p. 17716-20.

29. Lundberg, J.O., F.J. Larsen, and E. Weitzberg, *Supplementation with nitrate and nitrite salts in exercise: a word of caution.* J Appl Physiol, 2011. **111**(2): p. 616-7.

30. Jones, A.M., et al., *Reply to Lundberg, Larsen, and Weitzberg.* J Appl Physiol, 2011. **111**(2): p. 619.

31. Lauer, T., et al., *Age-dependent endothelial dysfunction is associated with failure to increase plasma nitrite in response to exercise.* Basic Res Cardiol, 2008. **103**(3): p. 291-7.

32. Rassaf, T., et al., *Nitric oxide synthase-derived plasma nitrite predicts exercise capacity.* Br J Sports Med, 2007. **41**(10): p. 669-73; discussion 673.

33. Dreissigacker, U., et al., *Positive correlation between plasma nitrite and performance during high-intensive exercise but not oxidative stress in healthy men.* Nitric Oxide, 2010. **23**(2): p. 128-35.

34. Larsen, F.J., et al., *Dietary nitrate reduces maximal oxygen consumption while maintaining work performance in maximal exercise.* Free Radic Biol Med, 2010. **48**(2): p. 342-7.

35. Bescos, R., et al., *Acute administration of inorganic nitrate reduces VO(2peak) in endurance athletes.* Med Sci Sports Exerc, 2011. **43**(10): p. 1979-86.

36. Larsen, F.J., et al., *Effects of dietary nitrate on oxygen cost during exercise.* Acta Physiol (Oxf), 2007. **191**(1): p. 59-66.

37. Bailey, S.J., et al., *Dietary nitrate supplementation reduces the O2 cost of low-intensity exercise and enhances tolerance to high-intensity exercise in humans.* J Appl Physiol, 2009. **107**(4): p. 1144-55.

38. Lansley, K.E., et al., *Dietary nitrate supplementation reduces the O2 cost of walking and running: a placebo-controlled study.* J Appl Physiol, 2011. **110**(3): p. 591-600.

39. Vanhatalo, A., et al., *Acute and chronic effects of dietary nitrate supplementation on blood pressure and the physiological responses to moderate-intensity and incremental exercise.* Am J Physiol Regul Integr Comp Physiol, 2010. **299**(4): p. R1121-31.

40. Lansley, K.E., et al., *Acute dietary nitrate supplementation improves cycling time trial performance.* Med Sci Sports Exerc, 2011. **43**(6): p. 1125-31.

41. Wilkerson, D.P., et al., *Influence of acute dietary nitrate supplementation on 50 mile time trial performance in well-trained cyclists.* Eur J Appl Physiol, 2012. **112**(12): p. 4127-34.

42. Christensen, P.M., M. Nyberg, and J. Bangsbo, *Influence of nitrate supplementation on VO(2) kinetics and endurance of elite cyclists.* Scand J Med Sci Sports, 2012.

43. Murphy, M., et al., *Whole beetroot consumption acutely improves running performance.* J Acad Nutr Diet, 2012. **112**(4): p. 548-52.

44. Burnley, M., et al., *Effects of prior heavy exercise on phase II pulmonary oxygen uptake kinetics during heavy exercise.* J Appl Physiol, 2000. **89**(4): p. 1387-96.

45. Wilkerson, D.P., N.J. Berger, and A.M. Jones, *Influence of hyperoxia on pulmonary O2 uptake kinetics following the onset of exercise in humans.* Respir Physiol Neurobiol, 2006. **153**(1): p. 92-106.

46. Jungersten, L., et al., *Both physical fitness and acute exercise regulate nitric oxide formation in healthy humans.* J Appl Physiol, 1997. **82**(3): p. 760-4.

47. Schena, F., et al., *Plasma nitrite/nitrate and erythropoietin levels in cross-country skiers during altitude training.* J Sports Med Phys Fitness, 2002. **42**(2): p. 129-34.

48. McConell, G.K., et al., *Skeletal muscle nNOS mu protein content is increased by exercise training in humans.* Am J Physiol Regul Integr Comp Physiol, 2007. **293**(2): p. R821-8.

49. McAllister, R.M. and M.H. Laughlin, *Vascular nitric oxide: effects of physical activity, importance for health.* Essays Biochem, 2006. **42**: p. 119-31.

50. Edwards, L.M., et al., *Endurance exercise training blunts the deleterious effect of high-fat feeding on whole body efficiency.* Am J Physiol Regul Integr Comp Physiol, 2011. **301**(2): p. R320-6.

51. Engan, H.K., et al., *Acute dietary nitrate supplementation improves dry static apnea performance.* Respir Physiol Neurobiol, 2012. **182**(2-3): p. 53-9.

52. Webb, A.J., et al., *Acute blood pressure lowering, vasoprotective, and antiplatelet properties of dietary nitrate via bioconversion to nitrite.* Hypertension, 2008. **51**(3): p. 784-90.

53. Hinkle, P.C., *P/O ratios of mitochondrial oxidative phosphorylation.* Biochim Biophys Acta, 2005. **1706**(1-2): p. 1-11.

54. Bose, S., et al., *Metabolic network control of oxidative phosphorylation: multiple roles of inorganic phosphate.* J Biol Chem, 2003. **278**(40): p. 39155-65.

55. Brown, G.C., *Control of respiration and ATP synthesis in mammalian mitochondria and cells.* Biochem J, 1992. **284 ( Pt 1)**: p. 1-13.

56. Larsen, F.J., et al., *Dietary inorganic nitrate improves mitochondrial efficiency in humans.* Cell Metab, 2011. **13**(2): p. 149-59.

57. Clerc, P., et al., *Nitric oxide increases oxidative phosphorylation efficiency.* J Bioenerg Biomembr, 2007. **39**(2): p. 158-66.

58. Gao, S., et al., *Docking of endothelial nitric oxide synthase (eNOS) to the mitochondrial outer membrane: a pentabasic amino acid sequence in the autoinhibitory domain of eNOS targets a proteinase K-cleavable peptide on the cytoplasmic face of mitochondria.* J Biol Chem, 2004. **279**(16): p. 15968-74.

59. Barclay, C.J., R.C. Woledge, and N.A. Curtin, *Energy turnover for Ca2+ cycling in skeletal muscle.* J Muscle Res Cell Motil, 2007. **28**(4-5): p. 259-74.

60. Walsh, B., et al., *Measurement of activation energy and oxidative phosphorylation onset kinetics in isolated muscle fibers in the absence of cross-bridge cycling.* Am J Physiol Regul Integr Comp Physiol, 2006. **290**(6): p. R1707-13.

61. Takaki, M., et al., *Sarcoplasmic reticulum Ca2+ pump blockade decreases O2 use of unloaded contracting rat heart slices: thapsigargin and cyclopiazonic acid.* J Mol Cell Cardiol, 1998. **30**(3): p. 649-59.

62. Evangelista, A.M., et al., *Direct regulation of striated muscle myosins by nitric oxide and endogenous nitrosothiols.* PLoS One, 2010. **5**(6): p. e11209.

63. Ishii, T., et al., *Inhibition of skeletal muscle sarcoplasmic reticulum Ca2+-ATPase by nitric oxide.* FEBS Lett, 1998. **440**(1-2): p. 218-22.

64. Larsen, F.J., et al., *Effects of dietary nitrate on blood pressure in healthy volunteers.* N Engl J Med, 2006. **355**(26): p. 2792-3.

65. Beall, C.M., et al., *Pulmonary nitric oxide in mountain dwellers.* Nature, 2001. **414**(6862): p. 411-2.

66. Erzurum, S.C., et al., *Higher blood flow and circulating NO products offset high-altitude hypoxia among Tibetans.* Proc Natl Acad Sci U S A, 2007. **104**(45): p. 17593-8.

67. Ge, R.L., et al., *Higher exercise performance and lower VO2max in Tibetan than Han residents at 4,700 m altitude.* J Appl Physiol, 1994. **77**(2): p. 684-91.

68. Duplain, H., et al., *Exhaled nitric oxide in high-altitude pulmonary edema: role in the regulation of pulmonary vascular tone and evidence for a role against inflammation.* Am J Respir Crit Care Med, 2000. **162**(1): p. 221-4.

69. Droma, Y., et al., *Positive association of the endothelial nitric oxide synthase gene polymorphisms with high-altitude pulmonary edema.* Circulation, 2002. **106**(7): p. 826-30.

70. Masschelein, E., et al., *Dietary nitrate improves muscle but not cerebral oxygenation status during exercise in hypoxia.* J Appl Physiol, 2012. **113**(5): p. 736-45.

71. Vanhatalo, A., et al., *Dietary nitrate reduces muscle metabolic perturbation and improves exercise tolerance in hypoxia.* J Physiol, 2011. **589**(Pt 22): p. 5517-28.

72. Kenjale, A.A., et al., *Dietary nitrate supplementation enhances exercise performance in peripheral arterial disease.* J Appl Physiol, 2011. **110**(6): p. 1582-91.

73. Boushel, R., et al., *Monitoring tissue oxygen availability with near infrared spectroscopy (NIRS) in health and disease.* Scand J Med Sci Sports, 2001. **11**(4): p. 213-22.

74. Grassi, B., et al., *Muscle oxygenation and pulmonary gas exchange kinetics during cycling exercise on-transitions in humans.* J Appl Physiol, 2003. **95**(1): p. 149-58.

75. Steiner, M.C., et al., *Bedside methods versus dual energy X-ray absorptiometry for body composition measurement in COPD.* Eur Respir J, 2002. **19**(4): p. 626-31.

76. Edwards, R.H., et al., *Human skeletal muscle function: description of tests and normal values.* Clin Sci Mol Med, 1977. **52**(3): p. 283-90.

77. O'Donnell, D.E., M. Lam, and K.A. Webb, *Measurement of symptoms, lung hyperinflation, and endurance during exercise in chronic obstructive pulmonary disease.* Am J Respir Crit Care Med, 1998. **158**(5 Pt 1): p. 1557-65.
